# Unmet needs for social support and diabetes-related distress among people living with type 2 diabetes in Thai Binh, Vietnam: a cross-sectional study

**DOI:** 10.1186/s12889-021-11562-6

**Published:** 2021-08-11

**Authors:** Diep Khong Thi, Bai Nguyen Xuan, Cuong Le Duc, Tine Gammeltoft, Jens Søndergaard, Dan Wolf Meyrowitsch, Ib Christian Bygbjerg, Jannie Nielsen

**Affiliations:** 1 Thai Binh University of Medicine and Pharmacy, 373 Ly Bon Street, Thai Binh city, Thai Binh Vietnam; 2 Department of Anthropology, University of Copenhagen, Øster Farimagsgade 5, DK-1353 Copenhagen K, Denmark; 3 Research Unit for General Practice, Department of Public Health, University of South Denmark, Odense, Denmark; 4 Global Health Section, Department of Public Health, University of Copenhagen, Øster Farimagsgade 5, 1014 Copenhagen K, Denmark; 5 Emory Global Diabetes Research Center, Hubert Department of Global Health, Rollins School of Global Health, Emory University, Atlanta, GA USA

**Keywords:** Diabetes-related distress, Type 2 diabetes, Unmet needs for social support, Vietnam

## Abstract

**Background:**

Diabetes-related distress (DRD) refers to negative emotional and affective experiences from daily demands of living with diabetes. People who received social support seem less likely to experience DRD. The prevalence of T2D in Vietnam is rapidly increasing. Yet, DRD and its association with social support have not been investigated. This study investigates DRD and how it is associated with unmet needs for social support in people with T2D in Thai Binh Province, Vietnam.

**Methods:**

A total of 806 people, age ≥ 40 years, treated for T2D at primary hospitals in Thai Binh Province, Vietnam, completed a questionnaire-based cross-sectional survey. DRD was self-reported, based on the Problem Areas In Diabetes scale 5 (PAID5). We assessed 6 types of unmet needs for social support from family/friends/community including: (i) Transport and company when visiting health facilities; (ii) Reminders to take medication; (iii) Purchase and preparation of food; (iv) Reminders to engage in physical exercise; (v) Emotional support; and (vi) Financial support. Multivariable logistic regression was used to model DRD as an outcome of each type of unmet need for social support, and as an outcome of the number of unmet needs for social support, adjusted for three sets of covariates.

**Results:**

In this study, 50.0% of people with T2D experienced DRD. Odds for DRD were higher among those who had any unmet need for social support. After adjustment for household economic status, only unmet needs for emotional and financial support were associated with higher odds ratios of DRD (OR = 2.59, CI95%: 1.19–5.63 and OR = 1.63, CI95%: 1.10–2.40, respectively). People who had ≥2 type of unmet need were not a higher risk of experiencing DRD as compared to those with no unmet need.

**Conclusions:**

Half of the people with T2D experienced DRD. The results suggest that having enough finances may decrease most needs for social support with the exception of emotional support. Thus, social support to financial and emotional of diabetes aspects may contribute to prevent or reverse DRD.

**Supplementary Information:**

The online version contains supplementary material available at 10.1186/s12889-021-11562-6.

## Background

Diabetes-related distress (DRD) refers to negative emotional and affective experiences from daily management of diabetes, such as feeling overwhelmed by the requirement of adhering to a specific diet, exercise, and medical care; or worrying about complications related to having diabetes [1, 2]. The concept of DRD was first presented in 1995 by Polonsky et al. who wanted to capture the emotional aspects of the psycho-social adjustment to diabetes [2]. The authors developed the Problem Areas in Diabetes Survey (PAID) including 20 items of diabetes-related distress e.g. anger, worries, and loneliness, which patients with diabetes had to rank on a Likert scale. Since then, PAID has been translated into and validated in various languages [3–7] and a short version including only 5 items has shown high sensitivity and specificity [8].

Experiences and perceptions of living with diabetes may vary across cultures. Still, results of a meta-analysis including 55 studies from high- and middle-income countries showed that more than one-third of people with type 2 diabetes (T2D) experienced DRD [1], while a study conducted in 17 countries, including India and China, found that 45% of the people with diabetes experienced DRD [9]. In South-East Asia, a cross-sectional study in Malaysia found that 50% of adults with T2D experienced DRD [10].

People experiencing DRD have a higher need for support, including social support [11]. In contrast, lower levels of DRD are seen in people who reported having social support to follow a specific diet, taking medicine, exercising, testing blood glucose levels, and handling feelings about diabetes [11, 12]. Further, due the broad range of emotional aspects of the psycho-social adjustment to diabetes included in the PAID and DDS measures, DRD has been shown to be associated with both demographic (age and gender) and socio-economic factors, reduced quality of life [13, 14], poor adherence to diabetes specific diet and medications [15], and diabetes duration [11, 12, 16–19].

From 2005 to 2012, the prevalence of people with diabetes in Vietnam increased with more than 200% [20]. In contrast, within the same period the prevalence of diabetes in the United States increased with 25% [21]. Of the people with diabetes in Vietnam, it is estimated that only 29% receive regular care for their diabetes at a health facility [22]. Conversely, self-care is common particularly in rural areas [22]. In other countries with a newly increasing burden of diabetes, social support to accessing health services, financial costs, preparing food, exercising, taking medicine, foot care, and encouragement about living with diabetes has been shown to positively influence management of diabetes [11, 16]. Yet, a qualitative component of this study from Thai Binh, Vietnam, suggest that social support may be insufficient: among people with diabetes, some did not involve family members in their diabetes as they did not want to burden them, while others felt that the social support they received was inadequate [23]. This is in line with an ethnographic component of this study showing that in Thai Binh, people do not want to burden others with negative emotions (Gammeltoft, Tine M: The Force of Love: Type II Diabetes in Vietnam as Tentatively Transformative Experience, forthcoming). Thus, in Vietnam, people with T2D may not receive or want to receive social support to manage diabetes, which could have compensated for the lack of treatment at health facilities. This may influence the experiences of living with diabetes and, thus, the emotional aspects of the psycho-social adjustment to diabetes. Therefore, we conducted a cross-sectional study in Thai Binh, Vietnam, to quantitively assess the proportion of people with T2D who experience DRD and investigate the association with unmet needs for social support.

## Methods

### Study population, setting, and recruitment

This cross-sectional survey collected self-reported questionnaire-based data, from December 2018 to February 2019, in people with T2D in Thai Binh Province, Vietnam, who had been diagnosed with T2D after the age of 40 years. Thai Binh Province is a rural province in the northern part of Vietnam and covers an area of 1542 km^2^, with a population of 1,860,447 people (2019). The province is divided into seven districts and one major city. This survey is part of the larger interdisciplinary project *VALID*, ‘Living Together with Chronic Disease: Informal Support for Diabetes Management in Vietnam’ [24].

In Vietnam, the health care system is divided into four levels: national, provincial, district, and communal level. For diabetes, primary treatment is provided by district hospitals; those with diabetes-related complications receive care at province and national hospitals. From the health authorities in Thai Binh Province, we obtained information about people with T2D (by district and commune of residence), who had received diabetes treatment at a district hospital. Two districts, Quynh Phu District in the northern part of Thai Binh and and Vu Thu District in the southern part, were randomly selected. In each district, we selected four communes: Two communes with the highest number of people with T2D, and a neighboring commune of each of the two selected communes, which was convenient for data collection.

**Fig. 1:**
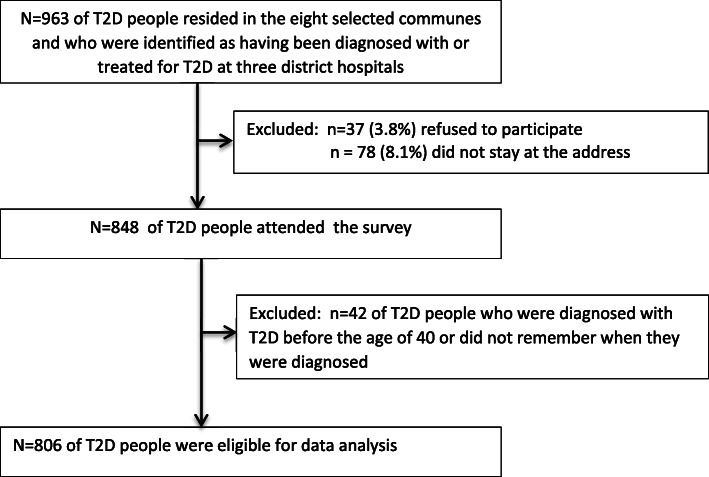
Diagram of participant recruiment

We recruited a total of 963 people, who resided in the eight selected communes and who were identified as having been diagnosed with or treated for T2D at a district hospitals. Among them, 37 (3.8%) refused to participate, and 78 (8.1%) did not stay at the address reported to the hospital or had moved away by the time of data collection. A total of 848 people participated in the cross-sectional survey. Among these, 42 individuals were excluded from the data analysis because they were diagnosed with T2D before the age of 40 or did not remember when they were diagnosed. A total of 806 participants were eligible for the analyses ‘Fig. 1’.

### Data collection

To collect data, we trained 16 village health workers from the 8 selected communes at a 2-day workshop followed by 1-day field-based training. To be invited to the study and to schedule the interviews, participants with T2D were contacted by mobile phone or via a personal visit. At the beginning of the interview, the outline of the project was explained, and persons agreeing to participate were asked to sign a consent form. After signing the consent form, the interviewer conducted the interview face-to-face, using a structured questionnaire developed for this study (eight domains, 176 questions, including 1) the study participants and their household (33 questions); 2) health status and use of health care services (46 questions); 3) life style regarding smoking, alcohol and exercise (6 questions); 4) homework - self-management of diabetes (19 questions); 5) informal social support (51 questions), including 6 questions related to unmet need for informal support); 6) sexual well-being and emotions (10 questions, including 5 questions related to distress); 7) use of smart phones, internet and other types of media (7 questions); and 8) comments to questionnaire, consent regarding further contact to support persons (4 questions) ‘Supplementary‘. In the present study, only domain 1, 2 and 5 were used. To maintain confidentiality, a village health worker could not collect data in her/his own commune and no other people than the interviewer and the participant could take part in the interview. Thus, the questionnaire contained questions that could be considered sensitive e.g. receiving support from family members and sexual health. All the participants were interviewed in their home.

### Outcome variable

To assess the experiences of DRD in the study population, we used the Problem Areas in Diabetes scale (PAID) [8]. We chose PAID as it focuses on emotional concerns related to coping with diabetes and diabetes complications, whereas other tools such as the Diabetes Distress Scale is more focused on motivational and behavioural problems associated with diabetes self-management and emotions related to physicians involved in diabetes treatment [25, 26]. We used the short PAID form (PAID5) as it has shown high sensitivity (94%) and specificity (89%) with the 20 item PAID and was chosen to minimize the interview burden on the participants. PAID5 was translated to Vietnamese, back-translated into English, and pilot-tested in a group of Vietnamese persons with diabetes to test the understanding, interpretation, and perception of the questions. The questions were also assessed in light of the project’s qualitative component where we found that the questions resonated with local framings of the problems. In other words, the topics that the questions address were covered in the ethnographic research on the project before being put to use quantitatively ([23]; Gammeltoft, Tine M: The Force of Love: Type II Diabetes in Vietnam as Tentatively Transformative Experience, forthcoming). PAID5 contains five questions: 1) Feeling scared when you think about living with diabetes; 2) Feeling depressed when you think about living with diabetes; 3) Worrying about the future and the possibility of serious complications; 4) Feeling that diabetes is taking up too much of your mental and physical energy every day; and 5) Coping with complications. Answers were scored at five levels: not a problem (0 points), minor problem (1 point), moderate problem (2 points), somewhat serious problem (3 points), and serious problem (4 points). The total score can range from zero to 20, and higher scores indicates more serious emotional problems [2]. To identify more severe experiences of DRD, a score of ≥33 on the 20-item PAID has been shown appropriate [27]. This score corresponds to a total score of ≥8 points on PAID5 [8], which we used to assess if people in Thai Binh had experienced DRD.

### Exposure variables

Social support is understood as help provided by family, friends, neighbors, relatives, and other informal care givers, and includes different areas of assistance, such as informational, emotional, companionship, and practical help [16–18]. An unmet need for social support was defined as inadequate amount of help received from informal sources in relation to daily management of diabetes [19]. We chose 6 areas of social support, which people with diabetes often receive from informal caregivers [16, 18] or have reported as unmet needs of support [28]. The 6 areas were: *(i)* Transport and company when visiting health facilities; (ii) Reminders to take medication; (iii) Purchase and preparation of food; (iv) Reminders to engage in physical exercise; (v) Emotional support; and (vi) Financial support related to any of the above-mentioned items. Answering options were: 1) I receive too little support: 2) I receive adequate support; and 3) I receive too much support. For each item, unmet need for the support was coded as a binary variable with “I receive too little support” coded 1, whereas the other answering options were coded as no unmet need for support (0). We also created a variable for the total number of unmet needs, categorized as 1, 2, 3, ≥4 unmet needs.

### Covariates

Demographic and socio-economic characteristics including gender (man, woman); age group (40–49 years, 50–59 years, 60–69 years, and ≥ 70 years); marital status (single, living with spouse, not living with spouse/divorced, or widowed); occupation (unemployed/stays at home, farmer, small trade business/worker/government employee/private company, and retired); size of household (one member, i.e. living alone, two members, 3–4 members, and ≥ 5 members); self-reported economic status of the household (poor, medium, and wealthy); self-reported physical health (poor, fair, and good); and duration of diabetes (< 5 years, 5–10 years, and > 10 years).

### Statistical analysis

The descriptive characteristics of people with T2D who experienced DRD are presented as frequencies. Chi-square tests were performed to compare the frequency of DRD by demographic, socio-economic, and health-related characteristics. A statistical significance level of *p* <  0.05 was used. We used logistic regression to model DRD as an outcome of each unmet need for social support to manage diabetes, and as an outcome of total number of types of unmet needs for social supports. All models were adjusted for age, gender, and household economic status (model 1). As a next step, we further adjusted for self-reported physical health (model 2). Lastly, we also included adjusted for number of co-residing household members, and duration of being diagnosed with diabetes. Results are presented as odd ratios (OR) with 95% confidence interval (95%CI). Data analyses were performed using SPSS (IBM Statistical Package for Social Science software) version 22.

## Results

### Demographic, social, economic, and health-related characteristics of people with diabetes-related distress

Characteristics of the people with T2D stratified by DRD status are presented in Table 1. Overall, the proportion of people with diabetes who had a PAID5 score equivalent to DRD was 50.0%. The proportion experiencing DRD differed by following determinants; gender (59.1% vs. 39.9% in women and men, respectively); marital status (single [68.4%], living with spouse [47.4%], not living with spouse/divorced [53.3%] and widowed [55.2%]); occupation (unemployed/stays at home [64.3%], farmer [57.4%], small trade business/worker/ government employee/private company [42.9%] and retired [36.8%]); self-report of economic situation of household (poor [74.7%], medium [47.5%] and wealthy [39.7%]); and self-report of physical health (poor [64.9%], fair [44.9%] and good [26.7%]) ‘Table 1’.

**Table 1:** Diabetes-related distress according to demographic, social, economic, and health-related characteristics

Characteristic	Overall	Diabetes-related distress^**1**^		***p*** value (χ^**2**^ test)
		YES Number (%)	NO Number (%)	
**All**		403 (50.0)	403 (50.0)	
**Gender**				
Men	381 (47.3)	152 (39.9)	229 (60.1)	< 0.001
Women	425 (52.7)	251 (59.1)	174 (40.9)	
**Age group**				
40–49	42 (5.2)	19 (45.2)	23 (54.8)	0.73
50–59	161 (20.0)	76 (47.2)	85 (52.8)	
60–69	357 (44.3)	180 (50.4)	177 (49.6)	
≥ 70	246 (30.5)	128 (52.0)	118 (48.0)	
**Marital status**				
Currently married and living with spouse	593 (73.6)	281 (47.4)	312 (52.6)	0.04
Single	38 (4.7)	26 (68.4)	12 (31.6)	
Not living with spouse /divorced	30 (3.7)	16 (53.3)	14 (46.7)	
Widowed	145 (18.0)	80 (55.2)	65 (44.8)	
**Occupation**				
Retired	258 (32.0)	95 (36.8)	163 (63.2)	< 0.001
Unemployed/stays at home	140 (17.4)	90 (64.3)	50 (35.7)	
Farmer	296 (36.7)	170 (57.4)	126 (42.6)	
Small trade/worker/government employee/private company	112 (13.9)	48 (42.9)	64 (57.1)	
**Size of household**				
5 members or more	218 (27.0)	111 (50.9)	107 (49.1)	0.08
3–4 members	181 (22.5)	82 (45.3)	99 (54.7)	
2 members	318 (39.5)	155 (48.7)	163 (51.3)	
1 member (living alone)	89 (11.0)	55 (61.8)	34 (38.2)	
**Economic situation of household (self-reported)**				
Wealthy	73 (9.1)	29 (39.7)	44 (60.3)	
Medium	638 (79.2)	303 (47.5)	335 (52.5)	< 0.001
Poor	95 (11.8)	71 (74.7)	24 (25.3)	
**Physical health (self-reported)**				
Excellent/Good	105 (13.0)	28 (26.7)	77 (73.3)	< 0.001
Fair	399 (49.5)	179 (44.9)	220 (55.1)	
Poor	302 (37.5)	196 (64.9)	106 (35.1)	
**Duration of diabetes**				
< 5 years	389 (48.3)	203 (52.2)	186 (47.8)	0.28
5–10 years	292 (36.2)	145 (49.7)	147 (50.3)	
> 10 years	125 (15.5)	55 (44.0)	70 (56.0)	

^1^. Diabetes-related distress is defined as stressful feelings associated with the challenges of managing diabetes and concerns related to diabetic complications

### Association between unmet needs for social support and diabetes-related distress

All of the six unmet needs were associated with higher odds of experiencing DRD ‘Table 2‘. After adjustment for age, gender and household economic status, higher odds of having DRD were observed if the person reported to have an unmet for emotional support or financial support (OR = 2.59, CI95%: 1.19–5.63 and OR = 1.63, CI95%: 1.10–2.40, respectively), while no associations were seen for the remaining types of unmet needs ‘Table 2’. For an unmet need for emotional support or financial support, additional adjustment for self-reported physical health attenuated the odds slightly (OR = 2.38, CI95%: 1.09–5.24 and OR = 1.49, CI95%: 1.00–2.22, respectively), while adjustment for household size, and diabetes duration attenuated the association between DRD and an unmet need for final support (OR = 1.45, CI95%: 0.97–2.17), but not between emotional support and DRD (OR = 2.36, CI95%: 1.07–5.23).

**Table 2:** Associations between unmet needs for social support and diabetes-related distress

Unmet needs for social support	Crude odds ratio (CI95%)	Model 1 Odds ratio (CI95%)	Model 2 Odds ratio (CI95%)	Model 3 Odds ratio (CI95%)
**Health care visits/transportation (*n*** **= 402)**				
No (ref) (*n* = 305)	1	1	1	1
Yes (*n* = 97)	**1.57 (1.11–2.22)**	1.22 (0.84–1.76)	1.14 (0.78–1.65)	1.10 (0.75–1.61)
**Remembering medication**				
No (ref) (*n* = 344)	1	1	1	1
Yes (*n* = 58)	1.53 (0.99–2.35)	1.27 (0.82–2.00)	1.14 (0.73–1.80)	1.14 (0.72–1.80)
**Purchasing and preparing food (n = 402)**				
No (ref) (*n* = 352)	1	1	1	1
Yes (*n* = 50)	**2.35 (1.40–2.93)**	1.62 (0.95–2.77)	1.53 (0.89–2.65)	1.48 (0.85–2.57)
**Remembering to doing exercise (*n*** **= 401)**				
No (ref) (*n* = 331)	1	1	1	1
Yes (*n* = 70)	**1.60 (1.07–2.38)**	1.36 (0.90–2.06)	1.21 (0.79–1.84)	1.17 (0.77–1.80)
**Emotional support (n = 402)**				
No (ref) (*n* = 371)	1	1	1	1
Yes (*n* = 31)	**3.65 (1.72–7.77)**	**2.59 (1.19–5.63)**	**2.38 (1.09–5.24)**	**2.36 (1.07–5.23)**
**Financial support with any of the above (n = 402)**				
No (ref) (*n* = 306)	1	1	1	1
Yes (*n* = 96)	**2.07 (1.43–2.99)**	**1.63 (1.10–2.40)**	**1.49 (1.00–2.22)**	1.45 (0.97–2.16)

Model 1: Adjusted for age, gender, household economic status

Model 2: Adjusted for age, gender, household economic status, physical health

Model 3: Adjusted for age, gender, household economic status, physical health, household size, duration of diabetes

### Associations between number of unmet needs for social support and diabetes-related distress

Bivariate analysis showed that there was no association between 1 unmet need and DRD, but those with 2 or more unmet needs had higher odds of having DRD: 2 unmet needs: OR = 1.77, CI95%: 1.01–3.13, 3 unmet needs: OR = 2.04, CI95%: 1.11–3.73, and 4–6 unmet needs: OR = 2.19, CI95%: 1.23–3.87 ‘Table 3‘. After adjusting for age, gender, and household economic status (Model 1); and self-reported health (Model 2); and number of co-residing household members and duration of diabetes (Model 3), the number of types of unmet needs was no longer associated with DRD ‘Table 3’.

**Table 3:** Associations between number of unmet needs for social support and diabetes-related distress

Number of unmet needs	Crude odds ratio (CI95%)	Model 1 Odd ratio (CI95%)	Model 2 Odds ratio (CI95%)	Model 3 Odds ratio (CI95%)
0 unmet need (*n* = 546) (reference)	1	1	1	1
1 unmet need (*n* = 95)	1.32 (0.85–2.04)	1.12 (0.72–1.77)	1.03 (0.65–1.64)	0.97 (0.60–1.54)
2 unmet needs (*n* = 55)	**1.77 (1.01–3.13)**	1.37 (0.76–2.47)	1.29 (0.71–2.36)	1.23 (0.67–2.25)
3 unmet needs (*n* = 49)	**2.04 (1.11–3.73)**	1.86 (0.99–3.47)	1.59 (0.84–3.00)	1.64 (0.86–3.10)
4–6 unmet needs (*n* = 57)	**2.19 (1.23–3.87)**	1.49 (0.82–2.71)	1.32 (0.72–2.41)	1.23 (0.66–2.27)

Model 1: Adjusted for age, gender, household economic status

Model 2: Adjusted for age, gender, household economic status, physical health

Model 3: Adjusted for age, gender, household economic status, physical health, household size, duration of diabetes

## Discussion

In this study, we found that 50.0% of adults with T2D in Thai Binh Province, Vietnam, experienced emotional concerns related to coping with diabetes and its complications at a level equivalent to DRD**.** The proportion of people experiencing DRD varied by gender, marital status, occupation, economic situation of household, and physical health. Those with unmet needs had higher odds of experiencing DRD than those reporting no unmet needs. The study also showed that ORs of experiencing DRD increased with increasing number of unmet needs. After adjustments for household economic status, only unmet needs for emotional support or financial support were associated with experiencing DRD.

In Thai Binh Province, Vietnam, 50% of people with T2D in Thai Binh experienced DRD, which is slightly higher than the 33–45% reported in two meta-analyses [1, 9]. However, DRD may be very context specific and thus not relate to the same aspects of diabetes or by felt or acknowledged similarly by people with diabetes living in different countries. Previous studies have shown that people with diabetes often want to get help and emotional support [14, 29]. Further, the more social support people with diabetes report to receive, including emotional support, the less likely they are to report experiencing DRD [30]. In Vietnam, ethnographic studies have shown that people tend to downplay feelings of distress or hide their emotions, particularly negative emotions, in an effort not to burden or disturb others (Gammeltoft, Tine M: The Force of Love: Type II Diabetes in Vietnam as Tentatively Transformative Experience, forthcoming; [31]). This does not mean that people are not aware of these emotions: they experience conflicting feelings with a desire to downplay and diminish the importance of the disease towards family members, but are constantly alert about how they need to manage their diabetes [23]. Such qualitative findings resonate with our findings of an unmet need for emotional support resulting in higher odds of experiencing DRD than other unmet needs. To which extent the people with T2D in our study downplay emotions is unknown, the unmet need for emotional support may be complicated by the mentioned desire to downplay emotions and it may be difficult for people with diabetes to seek emotional support. We found that 50% experienced DRD, which suggest that people with T2D in Vietnam may be more comfortable reporting the emotional aspects of living with diabetes to a ‘stranger’ during a one-to-one confidential interview than to e.g. family members.

Our findings showed that women had higher ORs of experiencing DRD than men, which are in line with studies from other countries [17–19]. In Vietnam, this gender difference may be partly explained by marriage and kinship practices: women in Vietnam often move far away from their own family to live with their husband’s family [31, 32]. This could lead to fewer relatives to share difficulties with, including health related problems. Previous research has shown that living far from one’s family can often generate feelings of loneliness and discomfort, making women more susceptible to distress [23, 31–33]. Moreover, in Vietnam women are, more often than men, in charge of housework, and care of children and old people and other family members [32], which may also add to the experiences of distress. Lastly, emotional violence against women is common in Vietnam (49.2%) [31], which could also contribute to the experiences of DRD in women [34].

Studies have shown that financial stress is associated with diabetes related costs such as medical expenses, travel expenses, and even daily activities, including costs for a particular diet [35, 36]. These results suggest that having enough finances may decrease most needs for social support. This is in line with our findings showing that the associations between experiencing DRD and four of the unmet needs were statistically significant before adjusting for household economic status. Though diabetes care in Vietnam is covered by medical insurance, people with diabetes still have to cover the costs for direct nonmedical items such as diabetes specific food or supplements, and transportation to health facilities providing treatment for diabetes, which are often far away as this treatment is only offered at district hospitals. A study from Vietnam estimated that these costs account for 14% of the cost of living with diabetes [29]. In addition, indirect cost related to diabetes such as workdays missed, reduced work productivity and reduced workforce participation because of health conditions accounted for 34.3% [29]. Accordingly, the unmet need for financial support was still associated with reporting DRD after adjustment for household economic status suggesting that those with low household economic status were also those reporting unmet needs for financial support. These findings are also supported by the qualitative study from Thai Binh, Vietnam, showing that people with T2D felt “guilty” when they felt that their diabetes status influenced their family’s incomes [23].

Of the people with T2D, 32% reported at least one unmet need for social support and crude analyses showed that all types of unmet needs were associated with experiencing DRD. Our findings showed that increasing number of unmet needs (≥2) increased the ORs of experiencing DRD, which is in line with result of studies from other countries showing that increasing social support and satisfaction with this support were associated with a lower proportion of people experiencing DRD [11, 12, 30]. Again, adjusting for household economic status diminished the association between unmet needs and experiencing DRD, indicating that financial worries may be a large contributor to experiences of DRD in Thai Binh, Vietnam.

In our study, unmet needs were self-reported. These reports may have been influenced by the Vietnamese culture of not wanting to burden others ([23]; Gammeltoft, Tine M: The Force of Love: Type II Diabetes in Vietnam as Tentatively Transformative Experience, forthcoming). Thus, a person may not be able to afford treatment or cook diabetes specific food, but will not consider such as unmet needs as they do not want to receive social support for these aspects. Further, the self-reports are also subjective meaning that a person with diabetes may objectively have e.g. too high blood glucose values, but if they do not perceive this as a problem, they would not need support for it. However, qualitative data shows that people with diabetes in Thai Binh are very cautious of how to prevent their diabetes from getting worse – though they may not all want social support for this (Gammeltoft, Tine M: The Force of Love: Type II Diabetes in Vietnam as Tentatively Transformative Experience, forthcoming). Given our findings of high odds for experiencing DRD in those with an unmet need for emotional support, social support in Vietnamese settings will likely have positively influences on DRD, but the social support may have to be initiated by the health system or a family member rather than by the person with diabetes.

A strength of this study is that it is the first quantitative study in Vietnam assessing the experiences of DRD in people with T2D, and of the association between unmet needs for social support and DRD. In addition, we had a large sample size. We also had a high response rate, which is in line with other studies from Vietnam [31, 37], However, the study had some limitations. First, participants were recruited from the list of outpatients at three district hospitals in two districts in Thai Binh province. Therefore, we did not include inpatients, or outpatients from province hospitals, or the national referral hospital, where people with more severe diabetes receive treatment. Therefore, the proportion of people experiencing DRD in our study may be lower than if these patients had been included. Second, unmet needs for social support were assessed using six questions, whereas the associations between unmet needs for social support and DRD may have had other dimensions, such as unmet needs for support for ordinary daily activities or unmet needs for guidance in performing self-management. Third, unmet needs were self-reported and due to the Vietnamese culture, Vietnamese people often tend to downplay or hide their emotions, particularly negative emotions, in an effort not to burden or disturb others. In other words, hiding one’s distress is a way of caring for others, and needs may have been underreported or not perceived as unmet needs. Fourth, to assess the experiences of DRD, we used the PAID5, which has not been validated in a Vietnamese population, which may have influenced our results. However, PAID5 is validated in neighboring countries such as Korea [38] and China [5]. Before using PAID5 in this study, we tested the PAID5 Vietnamese version in qualitative research and pilot-tested it in a group of Vietnamese people with diabetes to ensure the understanding, interpretation, and perception. Lastly, due to the purposively sampling, the results of this study may not be generalizable to Vietnamese people with T2D.

## Conclusions

This study from Thai Binh Province, Vietnam, found that 50% of people living with diabetes had a PAID5 score equivalent to experiencing DRD. All unmet needs for support from family and/or relatives increased the odds of experiencing DRD. After adjustment for household economic status, only unmet needs for emotional and financial support were associated with higher odds of experiencing DRD. Thus, our research suggests that enhanced social or financial support may contribute to prevent or reverse DRD.

## Supplementary Information

**Additional file 1.** Informal support for people living with diabetes.

## Data Availability

The datasets used and/or analyzed during the current study are available from the corresponding author on reasonable request.

## Abbreviations

DRD: Diabetes-related distress
T2D: type 2 diabetes
PAID5: Problem Areas In Diabetes scale 5
