# Supplementary material for: Unmet needs for social support and diabetes-related distress among people living with type 2 diabetes in Thai Binh, Vietnam: a cross-sectional study
Source: BMC Public Health. 2021 Aug 11;21:1532. doi: 10.1186/s12889-021-11562-6 (PMC8356389; doi:10.1186/s12889-021-11562-6)
Supplement: Supplementary file 1 — Additional file 1. Informal support for people living with diabetes. [file 12889_2021_11562_MOESM1_ESM.docx]

| Informal support for people living with diabetes  QUESTIONNAIRE 1  ‘  Name:  Village:  Commune:  Identification number (if known):  STUDY INFORMANT ID No:_______  DATE OF INTERVIEW (dd/mm/yyyy): …..…/……../……….   \| 000. RECORD THE TIME THE INTERVIEW BEGINS \| Hour [ ][ ] (24 h)  Minutes [ ][ ] \| \| --- \| --- \| \| 1. Name of interviewer 2. Name of supervisor \|  \| |
| --- | --- | --- | --- | --- |

| SECTION 1THE RESPONDENT AND HIS/HER HOUSEHOLD | | | |
| --- | --- | --- | --- |
| QUESTIONS & FILTERS | | CODING CATEGORIES | NOTES |
| If you don’t mind, I would like to start by asking you a little about yourself and the household that you live in.  Interviewer circles the selected option(s). | | |  |
| 101 | Gender of respondent | \| Male \| Female \| \| --- \| --- \| \| 0 \| 1 \| |  |
| 102 | Date and year of birth?  ‘Unknow’ date is marked as 99  ‘Unknow’ month is marked as 99  ‘Unknow’ year is marked as 9998  ‘Refused/no answer’ is marked as 9999 | DATE OF BIRTH: ___/_____/_____ (DD/MM/YYYY)  LUNAR DATE OF BIRTH: ___/_____/_____ (DD/MM/YYYY) |  |
| 102a: | If date of birth unknown:  How old are you? | \| AGE (YEARS) \| DON’T KNOW \| REFUSED/NO ANSWER \| \| --- \| --- \| --- \| \|  \| 888 \| 999 \| |  |
| 103 | Where did you grow up?  PROBE: Before age 18 where did you live longest? | \| THIS COMMUNE \| 1 \| \| --- \| --- \| \| ANOTHER COMMUNE \| 2 \| \| ANOTHER DISTRICT \| 3 \| \| ANOTHER PROVINCE OR CITY \| 4 \| \| DON’T KNOW/DON’T REMEMBER \| 8 \| \| REFUSED/NO ANSWER \| 9 \| |  |
| 104 | What is the highest level of education that you finished?  MARK HIGHEST LEVEL. | \| NEVER ATTENDED SCHOOL \| 0 \| \| --- \| --- \| \| PRIMARY SCHOOL \| 1 \| \| SECONDARY SCHOOL \| 2 \| \| HIGH SCHOOL \| 3 \| \| UNIVERSITY/COLLEGE \| 4 \| \| POSTGRADUATE \| 5 \| \| DON’T KNOW/DON’T REMEMBER \| 8 \| \| REFUSED/NO ANSWER \| 9 \| |  |
| 105 | What is your main current occupation? | \| UNEMPLOYED \| 0 \| \| --- \| --- \| \| STAY AT HOME WIFE/HUSBAND \| 1 \| \| FARMER \| 2 \| \| SMALL TRADE (SELF-BUSINESS) \| 3 \| \| WORKER \| 4 \| \| GOVERNMENT EMPLOYEE \| 5 \| \| PRIVATE COMPANY/ORGANISATION EMPLOYEE \| 6 \| \| RETIRED \| 7 \| \| STUDENT \| 8 \| \| REFUSED/NO ANSWER \| 9 \|   105_1: OTHER (SPECIFY)...................................................... | If retired  ⇒  105a/b, otherwise skip to 105d |
| 105a | If you are retired, when did you retire?  ‘Unknow’ date is marked as 99  ‘Unknow’ month is marked as 99  ‘Unknow’ year is marked as 9998  ‘Refused/no answer’ is marked as 9999 | YEAR OF RETIREMENT _____/_______/________ |  |
| 105b | If year unknown:  How old were you when your retired? | \| AGE (YEARS) \| DON’T KNOW \| REFUSED/NO ANSWER \| \| --- \| --- \| --- \| \|  \| 888 \| 999 \| |  |
| 105c | What was your main occupation prior to retirement? | \| WORKER \| 1 \| \| --- \| --- \| \| ARMY \| 2 \| \| POLICE \| 3 \| \| GOVERNMENT EMPLOYEE \| 4 \| \| PRIVATE COMPANY/ORGANISATION EMPLOYEE \| 5 \| \| OTHER \| 6 \| \| REFUSED/NO ANSWER \| 9 \|   105c_a: IF OTHER, SPECIFY:____________________________ |  |
| 105d | What is your household monthly income? | \| VND \| DON’T KNOW /DON’T REMEMBER \| KHÔNG TRẢ LỜI / TỪ CHỐI \| \| --- \| --- \| --- \| \|  \| 8 \| 9 \| |  |
| 106 | How would you yourself assess the economic situation of your household?  (Read up options) | \| POOR \| 1 \| \| --- \| --- \| \| NEAR POOR \| 2 \| \| MEDIUM \| 3 \| \| WEALTHY \| 4 \| \| DON'T KNOW \| 8 \| \| REFUSED/NO ANSWER \| 9 \| |  |
| 107 | How do local authorities assess the economic situation of your household?  (Read up options) | \| POOR \| 1 \| \| --- \| --- \| \| NEAR POOR \| 2 \| \| UNCATEGORIZED \| 3 \| \| DON'T KNOW \| 8 \| \| REFUSED/NO ANSWER \| 9 \| |  |
| 108 | Are you married? If yes, do you live together? | \| SINGLE \| 0 \| \| --- \| --- \| \| CURRENTLY MARRIED AND LIVING TOGETHER \| 1 \| \| CURRENTLY MARRIED BUT LIVING APART \| 2 \| \| LIVING TOGETHER, BUT NOT MARRIED \| 3 \| \| DIVORCED / SEPARATED \| 4 \| \| WIDOWED \| 5 \| \| REFUSED/NO ANSWER \| 9 \| | IF WIDOWED ⇒ 108A  OTHERWISE  ⇒ 109 |
| 108a | If you are widowed, which year did your spouse die?  ‘Unknow’ date is marked as 99  ‘Unknow’ month is marked as 99  ‘Unknow’ year is marked as 9998  ‘Refused/no answer’ is marked as 9999 | DATE/YEAR SPOUSE DIED: ___/_____/_____ (DD/MM/YYYY) | IF YEAR KNOWN ⇒ 109 |
| 108b | If year unknown:  How old were you when your spouse died? | \| AGE (YEARS) \| DON’T KNOW \| REFUSED/NO ANSWER \| \| --- \| --- \| --- \| \|  \| 888 \| 999 \| |  |
| 109 | Do you have children? | \| NO \| YES \| REFUSED/NO ANSWER \| \| --- \| --- \| --- \| \| 0 \| 1 \| 9 \| | IF NO  ⇒  110 |
| 109a | How many living sons do you have?  (only living children) | \| Number \| REFUSED/ NO ANSWER \| \| --- \| --- \| \|  \| 9 \| |  |
| 109b | How many living daughters do you have?  (only living children) | \| Number \| REFUSED/ NO ANSWER \| \| --- \| --- \| \|  \| 9 \| |  |
| 110 | What is the total number of persons living in your household?  (including domestic servants if they sleep 5 nights a week or more in the household and visitors if they have slept 5 nights or more a week in the household for the past 4 weeks) | \| Number \| REFUSED/ NO ANSWER \| \| --- \| --- \| \|  \| 9 \| | IF LIVES ALONE ⇒  111a |
| 110a | Who among these live in your household?  If “Yes”, put the number in the column under “YES”.  (Read up all options) | \|  \|  \| NO \| YES  (INSERT NUMBER) \| SOME OF THE TIME \| \| --- \| --- \| --- \| --- \| --- \| \| 110a_1 \| SPOUSE \| 0 \|  \| 99 \| \| 110a_2 \| MOTHER \| 0 \|  \| 99 \| \| 110a_3 \| FATHER \| 0 \|  \| 99 \| \| 110a_4 \| MOTHER-IN-LAW \| 0 \|  \| 99 \| \| 110a_5 \| FATHER-IN-LAW \| 0 \|  \| 99 \| \| 110a_6 \| GRANDPARENTS/ GRANDPARENTS-IN-LAW \| 0 \|  \| 99 \| \| 110a_7 \| SISTER \| 0 \|  \| 99 \| \| 110a_8 \| BROTHER \| 0 \|  \| 99 \| \| 110a_9 \| SISTER-IN-LAW \| 0 \|  \| 99 \| \| 110a_10 \| BROTHER-IN-LAW \| 0 \|  \| 99 \| \| 110a_11 \| DAUGHTER(S) \| 0 \|  \| 99 \| \| 110a_11 \| SON(S) \| 0 \|  \| 99 \| \| 110a_13 \| DAUGHTER(S)-IN-LAW \| 0 \|  \| 99 \| \| 110a_14 \| SON-IN-LAW \| 0 \|  \| 99 \| \| 110a_15 \| GRANDCHILDREN \| 0 \|  \| 99 \| \| 110a_16 \| GREAT-GRANDCHILDREN \| 0 \|  \| 99 \| \| 110a_17 \| OTHER \| 0 \|  \| 99 \| \| 110a_18 \| REFUSED/NO ANSWER \| 9 \|  \| 99 \|   110a_17a: IF OTHER, SPECIFY:____________________________  _ |  |
| 111a | Who is recorded as head of household in your household book? | \| I AM \| 1 \| \| --- \| --- \| \| MY SPOUSE \| 2 \| \| MY FATHER \| 3 \| \| MY MOTHER \| 4 \| \| MY FATHER-IN-LAW \| 5 \| \| MY MOTHER-IN-LAW \| 6 \| \| MY BROTHER \| 7 \| \| MY SISTER \| 8 \| \| MY SON \| 9 \| \| MY DAUGHTER \| 10 \| \| DAUGHTER-IN-LAW \| 11 \| \| SON-IN-LAW \| 12 \| \| Other \| 13 \| \| DONT KNOW \| 88 \| \| REFUSED/NO ANSWER \| 99 \|   111a_1a: IF OTHER, SPECIFY:_____________________________ | IF DON’T KNOW, => 110B, OTHERWISE => 111 |
| 111b | Who is taking the decisions in the household? | \| I AM \| 1 \| \| --- \| --- \| \| MY SPOUSE \| 2 \| \| MY FATHER \| 3 \| \| MY MOTHER \| 4 \| \| MY FATHER-IN-LAW \| 5 \| \| MY MOTHER-IN-LAW \| 6 \| \| MY BROTHER \| 7 \| \| MY SISTER \| 8 \| \| MY SON \| 9 \| \| MY DAUGHTER \| 10 \| \| MY DAUGHTER-IN-LAW \| 11 \| \| MY SON-IN-LAW \| 12 \| \| OTHER \| 13 \| \| DONT KNOW \| 88 \| \| REFUSED/NO ANSWER \| 99 \|   111b_1a: IF OTHER, SPECIFY:_____________________________ |  |
| 112 | Do you have any children who live **outside** your household? | \| NO \| YES \| REFUSED/NO ANSWER \| \| --- \| --- \| --- \| \| 0 \| 1 \| 9 \| | IF NO  ⇒  113 |
| 112a | If yes (children who live outside your household), where do they live?  (multiple answering options are allowed) | \| 112a_1 \|  \| THIS COMMUNE \| 1 \| \| --- \| --- \| --- \| --- \| \| 112a_2 \|  \| ANOTHER COMMUNE \| 2 \| \| 112a_3 \|  \| ANOTHER DISTRICT \| 3 \| \| 112a_4 \|  \| ANOTHER PROVINCE OR CITY \| 4 \| \| 112a_5 \|  \| DON’T KNOW/DON’T REMEMBER \| 8 \| \| 112a_6 \|  \| REFUSED/NO ANSWER \| 9 \| |  |
| 113 | Do you have any relatives who work as health professionals? | \| NO \| YES \| REFUSED/NO ANSWER \| \| --- \| --- \| --- \| \| 0 \| 1 \| 9 \| | IF NO  ⇒  114 |
| 113a | Which of your relative(s) work(s) as health professionals?  (Multiple answers allowed)  (Probe by asking of more than one relative is a health professional) | \|  \|  \| NO \| YES \| N/A \| \| --- \| --- \| --- \| --- \| --- \| \| 113a_1 \| MY SPOUSE \| 0 \| 1 \| 8 \| \| 113a_2 \| MY FATHER \| 0 \| 1 \| 8 \| \| 113a_3 \| MY MOTHER \| 0 \| 1 \| 8 \| \| 113a_4 \| MY FATHER-IN-LAW \| 0 \| 1 \| 8 \| \| 113a_5 \| MY MOTHER-IN-LAW \| 0 \| 1 \| 8 \| \| 113a_6 \| MY BROTHER \| 0 \| 1 \| 8 \| \| 113a_7 \| MY SISTER \| 0 \| 1 \| 8 \| \| 113a_8 \| MY SON \| 0 \| 1 \| 8 \| \| 113a_9 \| MY DAUGHTER \| 0 \| 1 \| 8 \| \| 113a_10 \| MY SON-IN-LAW \| 0 \| 1 \| 8 \| \| 113a_11 \| DAUGHTER-IN-LAW \| 0 \| 1 \| 8 \| \| 113a_12 \| MY NIECE \| 0 \| 1 \| 8 \| \| 113a_13 \| MY NEWPHEW \| 0 \| 1 \| 8 \| \| 113a_14 \| OTHER \| 0 \| 1 \| 8 \| \| 113a_15 \| DON’T KNOW \| 0 \| 1 \| 8 \| \| 113a_16 \| REFUSED/NO ANSWER \| 0 \| 1 \| 8 \|   113a_14a: IF OTHER; SPECIFY:___________________________ |  |
| 113b | Where do your relatives work?  (Multiple answers options allowed) | \| 113b_1 \| VILLAGE HEALTH WORKER \| 1 \| \| --- \| --- \| --- \| \| 113b_2 \| COMMUNE HEALTH STATION \| 2 \| \| 113b_3 \| DISTRICT HOSPITAL/HEALTH CENTRE \| 3 \| \| 113b_4 \| PROVINCIAL HOSPITAL/HEALTH CENTRE \| 4 \| \| 113b_5 \| NATIONAL-LEVEL HOSPITAL/HEALTH CENTRE \| 5 \| \| 113b_6 \| PHARMACY \| 6 \| \| 113b_7 \| TRADITIONAL MEDICINE \| 7 \| \| 113b_8 \| PRIVATE CLINIC AS EMPLOYEE \| 8 \| \| 113b_9 \| PRIVATE CLINIC AS OWNER \| 9 \| \| 113b_10 \| OTHER \| 10 \| \| 113b_11 \| DONT KNOW \| 88 \| \| 113b_12 \| REFUSED/NO ANSWER \| 99 \|   113b_10: IF OTHER , SPECIFY________________ |  |
| 114 | Do you have a health insurance? | \| NO \| YES \| REFUSED/ NO ANSWER \| \| --- \| --- \| --- \| \| 0 \| 1 \| 9 \| |  |
| 115 | Do you regularly attend a group, organization or association? | \| NO \| YES \| REFUSED/ NO ANSWER \| \| --- \| --- \| --- \| \| 0 \| 1 \| 9 \| |  |
| 115a | IF YES:  What kind of group, organization or association?  (multiple answering options are allowed)  IF NO, PROMPT:  Organizations like women’s or community groups, religious groups or political associations. | \|  \|  \| NO \| YES \| \| --- \| --- \| --- \| --- \| \| 115a_1 \| WOMEN’S UNION \| 0 \| 1 \| \| 115a_2 \| FARMERS’ UNION \| 0 \| 1 \| \| 115a_3 \| YOUTH UNION \| 0 \| 1 \| \| 115a_4 \| AGING UNION \| 0 \| 1 \| \| 115a_5 \| RELIGIOUS ORGANIZATION \| 0 \| 1 \| \| 115a_6 \| VETERANS’ ORGANIZATION \| 0 \| 1 \| \| 115a_7 \| COMMUNIST PARTY \| 0 \| 1 \| \| 115a_8 \| CULTURAL ASSOCIATION \| 0 \| 1 \| \| 115a_9 \| HEALTH CLUB (e.g. yoga, meditation) \| 0 \| 1 \| \| 115a_10 \| DIOXIN VICTIM ASSOCIATION \| 0 \| 1 \| \| 115a_11 \| OTHER \| 0 \| 1 \| \| 115a_12 \| REFUSED/NO ANSWER \| 0 \| 1 \|   115a_11a: IF OTHER, SPECIFY: ____________________________ |  |
| 116 | What is your religion/belief? | \| NO RELIGION \| 0 \| \| --- \| --- \| \| CHRISTIAN \| 1 \| \| BUDDHIST \| 2 \| \| OTHER \| 3 \| \| REFUSED/NO ANSWER \| 9 \|   116a: IF OTHER, SPECIFY:_______________________________ |  |
| 117 | For respondents born before 1960:  During the war against the United States, were you: | \| AT HOME \| 0 \| \| --- \| --- \| \| SOLDIER IN THE FRONTLINE \| 1 \| \| SOLDIER BEHIND THE FRONTLINE \| 2 \| \| VOLUNTEERS IN THE FRONTLINE \| 3 \| \| REFUSED/NO ANSWER \| 9 \| | IF NOT ACTIVE DURING  THE WAR:  ⇒ SECTION 2 |
| 117a | If you were active in the war, were you injured physically? | \| NO \| YES \| REFUSED/ NO ANSWER \| \| --- \| --- \| --- \| \| 0 \| 1 \| 9 \| |  |

| **SECTION 2**  **HEALTH AND USE OF HEALTH CARE SERVICES** | | | | | | | | | | | | | | | | | | | | | | | | | | | | | | | | | | | | | | | | | |
| --- | --- | --- | --- | --- | --- | --- | --- | --- | --- | --- | --- | --- | --- | --- | --- | --- | --- | --- | --- | --- | --- | --- | --- | --- | --- | --- | --- | --- | --- | --- | --- | --- | --- | --- | --- | --- | --- | --- | --- | --- | --- |
| I would now like to ask a few questions about your health and use of health services. | | | | | | | | | | | | | | | | | | | | | | | | | | | | | | | | | | | | | | | | | |
| 201 | In general, how would you describe your overall physical health?  (Read up options) | | \| EXCELLENT \| 1 \| \| --- \| --- \| \| GOOD \| 2 \| \| FAIR \| 3 \| \| POOR \| 4 \| \| VERY POOR \| 5 \| \| REFUSED/NO ANSWER \| 9 \| | | | | | | | | | | | | | | | | | | | | | | | | | | | | | | | | | | | | | |  |
| 202 | In general, how would you describe your overall mental health?  (Read up options) | | \| EXCELLENT \| 1 \| \| --- \| --- \| \| GOOD \| 2 \| \| FAIR \| 3 \| \| POOR \| 4 \| \| VERY POOR \| 5 \| \| REFUSED/NO ANSWER \| 9 \| | | | | | | | | | | | | | | | | | | | | | | | | | | | | | | | | | | | | | |  |
| 203 | Have you ever been diagnosed with the following medical conditions (other than diabetes)? | | \|  \|  \| NO \| YES \| REFUSED/ NO ANSWER \| \| --- \| --- \| --- \| --- \| --- \| \| 203_1 \| HYPERTENSION \| 0 \| 1 \| 9 \| \| 203_2 \| EPILEPSY \| 0 \| 1 \| 9 \| \| 203_3 \| DEPRESSION (Please probe) \| 0 \| 1 \| 9 \| \| 203_4 \| TUBERCULOSIS \| 0 \| 1 \| 9 \| \| 203_5 \| LIVER AND KIDNEY DISEASE \| 0 \| 1 \| 9 \| \| 203_6 \| BONE AND JOINT PROBLEM \| 0 \| 1 \| 9 \| \| 203_7 \| Cardio-vascular disease \| 0 \| 1 \| 9 \| \| 203_8 \| ANY OTHER CHRONIC DISEASE \| 0 \| 1 \| 9 \|   203_8a: IF OTHER SPECIFY: _______________________________  _______________________________________________________ | | | | | | | | | | | | | | | | | | | | | | | | | | | | | | | | | | | | | |  |
| 204 | **When** did you get the diagnosis of diabetes?  Please specify day, month and year: ‘Unknow’ date is marked as 99  ‘Unknow’ month is marked as 99  ‘Unknow’ year is marked as 9998  ‘Refused/no answer’ is marked as 9999 | | ___/_____/_____ (DD/MM/YYYY) | | | | | | | | | | | | | | | | | | | | | | | | | | | | | | | | | | | | | | IF DATE KNOWN ⇒  205 |
| 204a | How old were you when were diagnosed with diabetes? | | \| AGE (YEARS) \| DON’T KNOW \| REFUSED/NO ANSWER \| \| --- \| --- \| --- \| \|  \| 888 \| 999 \| | | | | | | | | | | | | | | | | | | | | | | | | | | | | | | | | | | | | | |  |
| 204b | **IF date unknow, ask:** | | \| MORE THAN 8 YEARS AGO \| 1 \| \| --- \| --- \| \| 3-8 YEARS AGO \| 2 \| \| 1-3 YEARS AGO \| 3 \| \| LESS THAN 1 YEAR AGO \| 4 \| \| LESS THAN 1 MONTH AGO \| 5 \| \| DON’T KNOW \| 8 \| \| REFUSED/NO ANSWER \| 9 \| | | | | | | | | | | | | | | | | | | | | | | | | | | | | | | | | | | | | | |  |
| 205 | **Where** did you get the diagnosis of diabetes? | | \| NATIONAL-LEVEL HOSPITAL \| 1 \| \| --- \| --- \| \| PROVINCIAL HOSPITAL \| 2 \| \| DISTRICT HOSPITAL \| 3 \| \| PRIVATE HOSPITAL/CLINIC \| 4 \| \| OTHER (SPECIFY) \| 5 \| \| DON’T KNOW/DON’T REMEMBER \| 8 \| \| REFUSED/NO ANSWER \| 9 \|   205a: IF OTHER, SPECIFY: ________________________________ | | | | | | | | | | | | | | | | | | | | | | | | | | | | | | | | | | | | | |  |
| 206 | **Where** do you usually go for check-up and treatment of diabetes?  (multiple answering options are allowed) | | \| NATIONAL-LEVEL HOSPITAL \| 1 \| \| --- \| --- \| \| PROVINCIAL HOSPITAL \| 2 \| \| DISTRICT HOSPITAL \| 3 \| \| PRIVATE HOSPITAL/CLINIC \| 4 \| \| COMMUNE HEALTH STATION \| 5 \| \| OTHER (SPECIFY) \| 6 \| \| REFUSED/NO ANSWER \| 9 \|   206_1a: IF OTHER, SPECIFY: ________________________________ | | | | | | | | | | | | | | | | | | | | | | | | | | | | | | | | | | | | | |  |
| 206a | **How often** do you usually go for check-up and treatment of diabetes? | | \| SEVERAL TIMES PER MONTH \| 1 \| \| --- \| --- \| \| EVERY MONTH \| 2 \| \| EVERY 3 MONTHS \| 3 \| \| EVERY 6 MONTHS \| 4 \| \| IRREGULARLY \| 5 \| \| OTHER \| 6 \| \| DON'T KNOW \| 8 \| \| REFUSED/NO ANSWER \| 9 \|   206a_1a: IF OTHER, SPECIFY: ________________________________ | | | | | | | | | | | | | | | | | | | | | | | | | | | | | | | | | | | | | |  |
| 206b | **Who** decides how often you should go for diabetes check up/treatment? | | \| I MYSELF \| 1 \| \| --- \| --- \| \| HEALTH STAFF \| 2 \| \| RELATIVES \| 3 \| \| OTHER \| 4 \| \| REFUSED/NO ANSWER \| 9 \|   206b_1a: IF OTHER, SPECIFY: ________________________________ | | | | | | | | | | | | | | | | | | | | | | | | | | | | | | | | | | | | | |  |
| 206c | According to your health provider, how often should you come for diabetes check-ups? | | \| SEVERAL TIMES PER MONTH \| 1 \| \| --- \| --- \| \| EVERY MONTH \| 2 \| \| EVERY 3 MONTHS \| 3 \| \| EVERY 6 MONTHS \| 4 \| \| IRREGULARLY \| 5 \| \| OTHER \| 6 \| \| REFUSED/NO ANSWER \| 9 \|   206c_1a: IF OTHER, SPECIFY: ________________________________ | | | | | | | | | | | | | | | | | | | | | | | | | | | | | | | | | | | | | |  |
| 206d | How often do you manage to attend the diabetes check-ups recommended by your health provider?  (Read up option 1-3) | | \| RARELY \| 1 \| \| --- \| --- \| \| SOMETIMES \| 2 \| \| ALWAYS \| 3 \| \| REFUSED/NO ANSWER \| 9 \| | | | | | | | | | | | | | | | | | | | | | | | | | | | | | | | | | | | | | |  |
| 206e | How long time does it take you to get to the health facility where you most often receive treatment? | | \| < 15 MINUTES \| 1 \| \| --- \| --- \| \| 15 MINUTES TO < 30 MINUTES \| 2 \| \| ≥ 30 TO < 1 HOUR \| 3 \| \| ≥ 1 HOUR TO < 2 HOURS \| 4 \| \| ≥ 2 HOURS \| 5 \| \| REFUSED/NO ANSWER \| 9 \| | | | | | | | | | | | | | | | | | | | | | | | | | | | | | | | | | | | | | |  |
| 207 | DOES THE PATIENT HAVE INFORMATION ABOUT THEIR LAST CHECKUP + BLOOD GLUCOSE MEASURE IN THE BOOKLET/PAPER FROM HOSPITAL | | \| NO \| YES \| \| --- \| --- \| \| 0 \| 1 \| | | | | | | | | | | | | | | | | | | | | | | | | | | | | | | | | | | | | | | If no, move to 207c |
| 207a | IF YES Blood Glucose value  If blood glucose level indicate missing: 999.99 | | __________________ | | | | | | | | | | | | | | | | | | | | | | | | | | | | | | | | | | | | | |  |
| 207b | If date missing: 99/99/9999 | | ___/_____/_____ (DD/MM/YYYY) | | | | | | | | | | | | | | | | | | | | | | | | | | | | | | | | | | | | | |  |
| 207c | IF NO, (207), if there are no information in booklet/from hospital: DO YOU REMEMBER WHEN YOU LAST TIME WENT FOR CHECK-UP? | | \| LESS THAN 1 WEEK AGO \| 1 \| \| --- \| --- \| \| FROM 1 WEEK TO 4 WEEKS AGO \| 2 \| \| FROM 1 MONTHS TO 3 MONTHS \| 3 \| \| MORE THAN 3 MONTHS AGO \| 4 \| \| DON’T REMEMBER \| 9 \| | | | | | | | | | | | | | | | | | | | | | | | | | | | | | | | | | | | | | | For interviewer |
| 207d | IF NO (207), can you recall your last measured? (ask about value)  If don’t remember blood glucose value indicate missing: 999.99 | | (write value) | | | | | | | | | | | | | | | | | | | | | | | | | | | | | | | | | | | | | |  |
| 208 | What type of medication do you take for your diabetes? | | | | | | | | | | | | | | | | | | | | | | | | | | | | | | | | | | | | | | | |  |
|  |  | NO MEDICATION | 0 | | | | | | | | | | |  | | | | | | | | | | | | | | | | | | | | | | | | | | |  |
|  |  | ORAL MEDICATION | 1 | | | | | | | | | | |  |  |  |  |  |  |  |  |  |  |  |  |  |  |  |  |  |  |  |  |  |  |  |  |  |  |  |  |
|  |  | INSULIN/ INJECTIONS | 2 | | | | | | | | | | |  |  |  |  |  |  |  |  |  |  |  |  |  |  |  |  |  |  |  |  |  |  |  |  |  |  |  |  |
|  |  | BOTH | 3 | | | | | | | | | | |  |  |  |  |  |  |  |  |  |  |  |  |  |  |  |  |  |  |  |  |  |  |  |  |  |  |  |  |
|  |  | REFUSED | 9 | | | | | | | | | | |  |  |  |  |  |  |  |  |  |  |  |  |  |  |  |  |  |  |  |  |  |  |  |  |  |  |  |  |
| 208a | Specify all types of medication prescribed in the patients ’patient-book’ AT LAST CHECK-UP:  1.__________________________________________________________  2.__________________________________________________________  3.__________________________________________________________  4.__________________________________________________________  5.__________________________________________________________ | | | | | | | | | | | | | | | | | | | | | | | | | | | | | | | | | | | | | | | |  |
| 208b | In the past 4 weeks, how often did you take the medications the doctor prescribed **for your diabetes**:  (Read up all options) | | | | | | | | | | | | | | | | | | | | | | | | | | | | | | | | | | | | | | | | IF 3 move to 208d |
|  | \| NO \| 0 \| \| --- \| --- \| \| YES - ONCE OR TWICE PER MONTH \| 1 \| \| YES - A FEW TIMES WEEKLY \| 2 \| \| EVERY DAY \| 3 \| \| DONT KNOW \| 8 \| \| REFUSED NO ANSWER \| 9 \| | | | | | | | | | | | | | | | | | | | | | | | | | | | | | | | | | | | | | | | |  |
| 208c | Why didn’t you take the medication the doctor prescribed for your diabetes? (MULTIPLE CHOICES) | | | | | | | | | | | | | | | | | | | | | | | | | | | | | | | | | | | | | | | |  |
|  |  |  | | | | | | | | NO | | | | | | | | YES | | | | | | | | DONT KNOW | | | | | | | | | REFUSED/  NO ANSWER | | | | | |  |
|  | 208c_1 | CONTROL BLOOD GLUCOSE LEVEL BY DIET | | | | | | | | 0 | | | | | | | | 1 | | | | | | | | 8 | | | | | | | | | 9 | | | | | |  |
|  | 208c_2 | THINK THAT BLOOD GLUCOSE IS NOT HIGH | | | | | | | | 0 | | | | | | | | 1 | | | | | | | | 8 | | | | | | | | | 9 | | | | | |  |
|  | 208c_3 | WORRY ABOUT NEGATIVE EFFECTS OF DIABETIC MEDICINE | | | | | | | | 0 | | | | | | | | 1 | | | | | | | | 8 | | | | | | | | | 9 | | | | | |  |
|  | 208c_4 | OUT OF MEDICINE, NO TIME TO TAKE FROM HOSPITAL | | | | | | | | 0 | | | | | | | | 1 | | | | | | | | 8 | | | | | | | | | 9 | | | | | |  |
|  | 208c_5 | OUT OF MEDICINE, NO MONEY TO BUY OR NO TIME TO BUY | | | | | | | | 0 | | | | | | | | 1 | | | | | | | | 8 | | | | | | | | | 9 | | | | | |  |
|  | 208c_6 | OTHER | | | | | | | | 0 | | | | | | | | 1 | | | | | | | | 8 | | | | | | | | | 9 | | | | | |  |
|  | 208c_6a | IF OTHER, SPECIFY:_________________________________________________________________ | | | | | | | | | | | | | | | | | | | | | | | | | | | | | | | | | | | | | | |  |
| 208d | SPECIFY MEDICATION YOU TAKE FOR YOUR DIABETES THAT ARE NOT PRESCRIBED BY THE DOCTOR?  1.__________________________________________________________  2.__________________________________________________________  3.__________________________________________________________  4.__________________________________________________________  5.__________________________________________________________ | | | | | | | | | | | | | | | | | | | | | | | | | | | | | | | | | | | | | | | |  |
| 208e | SPECIFY MEDICATION YOU TAKE IN THE PAST 4 WEEK  (IN CASE, PATIENTS DON’T KEEP THE “PATIENTS-BOOK” AND THEY ALSO DON’T KNOW THE SOURCE OF THE MEDICATION THEY TOOK)  1.__________________________________________________________  2.__________________________________________________________  3.__________________________________________________________  4.__________________________________________________________  5.________________________________________________________ | | | | | | | | | | | | | | | | | | | | | | | | | | | | | | | | | | | | | | | |  |
| 208f | In the past 4 weeks, have you taken any of the following herbs **for your diabetes**:  (Read up all options)  FOR EACH **“YES” PROBE:**  How often? Once or twice, a few times weekly or daily? | | | | | | | | | | | | | | | | | | | | | | | | | | | | | | | | | | | | | | | |  |
|  | VIETNAMESE HERB/CHINESE HERB | | | NO | | | | | YES - ONCE OR TWICE | | | | | | | | YES -  A FEW TIMES WEEKLY | | | | EVERY DAY | | | | | | DONT KNOW | | | | | | | REFUSED NO ANSWER | | | | | | |  |
|  | 208f_1 | VIETNAMESE HERBS ( LEAF : GYMNEMA SYLVESTRE, GUAVA, MANGO…) | | 0 | | | | | 1 | | | | | | | | 2 | | | | 3 | | | | | | 8 | | | | | | | 9 | | | | | | |  |
|  | 208f_2 | CHINESE HERBS | | 0 | | | | | 1 | | | | | | | | 2 | | | | 3 | | | | | | 8 | | | | | | | 9 | | | | | | |  |
|  | 208f_3 | FUNCTIONAL FOODS | | 0 | | | | | 1 | | | | | | | | 2 | | | | 3 | | | | | | 8 | | | | | | | 9 | | | | | | |  |
|  | 208f_3a | If Yes, (1,2 or 3) for 208e_3, please specifiy: | | _____________________________________ | | | | | | | | | | | | | | | | | | | | | | | | | | | | | | | | | | | | |  |
| 209 | In the past 4 weeks, have you taken any other medications?:  (Read up all options)  FOR EACH **“YES” PROBE:**  How often? Once or twice, a few times weekly or daily? | | | | | | | | | | | | | | | | | | | | | | | | | | | | | | | | | | | | | | | |  |
|  | **OTHER MEDICINES**  The same medication can be taken for different conditions | | NO | | YES - ONCE OR TWICE | | | | | | | YES -  A FEW TIMES WEEKLY | | | | | | | EVERY DAY | | | | | | | | | | DONT KNOW | | | | | | | REFUSED NO ANSWER | | | | |  |
|  | 209_1 | FOR HYPERTENTION  _________ | 0 | | 1 | | | | | | | 2 | | | | | | | 3 | | | | | | | | | | 8 | | | | | | | 9 | | | | |  |
|  | 209_2 | FOR EYES | 0 | | 1 | | | | | | | 2 | | | | | | | 3 | | | | | | | | | | 8 | | | | | | | 9 | | | | |  |
|  | 209_3 | FOR KIDNEY/LIVER | 0 | | 1 | | | | | | | 2 | | | | | | | 3 | | | | | | | | | | 8 | | | | | | | 9 | | | | |  |
|  | 209_4 | FOR NERVE | 0 | | 1 | | | | | | | 2 | | | | | | | 3 | | | | | | | | | | 8 | | | | | | | 9 | | | | |  |
|  | 209_5 | FOR OTHER DISEASE | 0 | | 1 | | | | | | | 2 | | | | | | | 3 | | | | | | | | | | 8 | | | | | | | 9 | | | | |  |
| 210 | Do you use insulin? | | NO | | | | | | | | | | | 0 | | | | | |  | | | | | | | | | | | | | | | | | | | | | IF 0, 8,9 🡪 212 |
|  |  |  | YES, SELF-FILLING UP INSULIN, SELF- INJECTED | | | | | | | | | | | 1 | | | | | |  |  |  |  |  |  |  |  |  |  |  |  |  |  |  |  |  |  |  |  |  |  |
|  |  |  | YES, NO - FILLING UP INSULIN, SELF- INJECTED | | | | | | | | | | | 2 | | | | | |  |  |  |  |  |  |  |  |  |  |  |  |  |  |  |  |  |  |  |  |  |  |
|  |  |  | YES, BE INJECTED BY OTHERS | | | | | | | | | | | 3 | | | | | |  |  |  |  |  |  |  |  |  |  |  |  |  |  |  |  |  |  |  |  |  |  |
|  |  |  | REFUSED/ NO ANSWER | | | | | | | | | | | 9 | | | | | |  |  |  |  |  |  |  |  |  |  |  |  |  |  |  |  |  |  |  |  |  |  |
|  |  | | 210_a: IF OTHER, SPECIFY:____________________________ | | | | | | | | | | | | | | | | | | | | | | | | | | | | | | | | | | | | | |  |
| 211 | Who helped you to use insulin? | | \|  \|  \| NO \| YES \| N/A \| \| --- \| --- \| --- \| --- \| --- \| \| 210_1 \| MYSELF \| 0 \| 1 \| 8 \| \| 210_2 \| SPOUSE \| 0 \| 1 \| 8 \| \| 210_3 \| DAUGHTER \| 0 \| 1 \| 8 \| \| 210_4 \| SON \| 0 \| 1 \| 8 \| \| 210_5 \| DAUGHTER-IN-LAW \| 0 \| 1 \| 8 \| \| 210_6 \| SON-IN-LAW \| 0 \| 1 \| 8 \| \| 210_7 \| VILLAGE HEATH WORKER \| 0 \| 1 \| 8 \| \| 210_8 \| HEALTH STAFF IN HEALTH STATION \| 0 \| 1 \| 8 \| \| 210_9 \| OTHER PERSON WITH DIABETES \| 0 \| 1 \| 8 \| \| 210_10 \| OTHER \| 0 \| 1 \| 8 \|   210_10a: Other, SPECIFY: _________________________________ | | | | | | | | | | | | | | | | | | | | | | | | | | | | | | | | | | | | | |  |
| 212 | DO YOU USE MEDICINE IN HEALTH INSURANCE OR BUY YOURSELF? (Multiple options allowed) | | | | | | | | | | | | | | | | | | | | | | | | | | | | | | | | | | | | | | | |  |
|  |  |  | NO | | | | | | | | | | | | | | | YES | | | | | | DONT KNOW | | | | | | | | | | | | | REFUSED/NO ANSWER | | | | If ONESELF -> 212c |
|  | 212_1 | HEALTH INSURANCE | 0 | | | | | | | | | | | | | | | 1 | | | | | | 8 | | | | | | | | | | | | | 9 | | | |  |
|  | 212_2 | ONESELF | 0 | | | | | | | | | | | | | | | 1 | | | | | | 8 | | | | | | | | | | | | | 9 | | | |  |
| 212a | If you only use medicine in health insurance, do you have to spend money for diabetes medication? | | \| No \| 0 \| \| --- \| --- \| \| Yes \| 1 \| | | | | | | | | | | | | | | | | | | | | | | | | | | | | | | | | | | | | | | If No,  ->214 |
| 212b | If you have to spend money for diabetic medicine in health insurance, how much do you spend on medicaion per month? | | \| VND \| DON’T/DON’T REMEMBER \| REFUSED/NO ANSWER \| \| --- \| --- \| --- \| \|  \| 8 \| 9 \| | | | | | | | | | | | | | | | | | | | | | | | | | | | | | | | | | | | | | |  |
| 212c | If you have to buy medication by yourself. How much do you spend on diabetes medication per month? | | \| VND \| DON’T/DON’T REMEMBER \| REFUSED/NO ANSWER \| \| --- \| --- \| --- \| \|  \| 8 \| 9 \| | | | | | | | | | | | | | | | | | | | | | | | | | | | | | | | | | | | | | |  |
| 213 | Why do you use diabetes medicine NOT COVERED (PAID) BY the health insurance? | | | | | | | | | | | | | | | | | | | | | | | | | | | | | | | | | | | | | | | |  |
|  |  | | NO | | | | | | | | | | YES | | | | | | | | | | | DONT KNOW | | | | | | | REFUSED/NO ANSWER | | | | | | | | | |  |
|  | 213_1 | DON’T TRUST THE QUALITY OF INSURANCE MEDICINE | 0 | | | | | | | | | | 1 | | | | | | | | | | | 8 | | | | | | | 9 | | | | | | | | | |  |
|  | 213_2 | THINK THE INSURANCE MEDICATION IS LESS EFFECTIVE | 0 | | | | | | | | | | 1 | | | | | | | | | | | 8 | | | | | | | 9 | | | | | | | | | |  |
|  | 213_3 | TO PICK UP THE INSURANCE MEDICINE TAKE TOO MUCH TIME | 0 | | | | | | | | | | 1 | | | | | | | | | | | 8 | | | | | | | 9 | | | | | | | | | |  |
|  | 213_4 | INSURANCE MEDICINE HAVE MORE SIDE EFFECTS | 0 | | | | | | | | | | 1 | | | | | | | | | | | 8 | | | | | | | 9 | | | | | | | | | |  |
|  | 213_5 | OTHERS ENCOURAGE ME TO TAKE IT | 0 | | | | | | | | | | 1 | | | | | | | | | | | 8 | | | | | | | 9 | | | | | | | | | |  |
|  | 213_6 | GIVEN BY OTHER PEOPLE | 0 | | | | | | | | | | 1 | | | | | | | | | | | 8 | | | | | | | 9 | | | | | | | | | |  |
|  | 213_7 | OTHER | 0 | | | | | | | | | | 1 | | | | | | | | | | | 8 | | | | | | | 9 | | | | | | | | | |  |
|  |  | 213_7a: IF OTHER, SPECIFY:_____________________________________________________________ | | | | | | | | | | | | | | | | | | | | | | | | | | | | | | | | | | | | | | |  |
| 214 | The next questions are related to common problems that may have bothered you in the past 4 weeks. If you had the problem **in the past 4 weeks,** answer **yes.** If you have not had the problem in the past 4 weeks, answer **no.** | | | | | | | | | | | | | | | | | | | | | | | | | | | | | | | | | | | | | | | |  |
|  |  | | | | | NO | | | | | | | | | | | YES | | | | | | | | DONT KNOW | | | | | | | | | | | | | REFUSED/NO ANSWER | | |  |
|  | 214_1 | Do you often have headaches? | | | | 0 | | | | | | | | | | | 1 | | | | | | | | 8 | | | | | | | | | | | | | 9 | | |  |
|  | 214_2 | Is your appetite poor? | | | | 0 | | | | | | | | | | | 1 | | | | | | | | 8 | | | | | | | | | | | | | 9 | | |  |
|  | 214_3 | Do you sleep badly? | | | | 0 | | | | | | | | | | | 1 | | | | | | | | 8 | | | | | | | | | | | | | 9 | | |  |
|  | 214_4 | Are you easily frightened? | | | | 0 | | | | | | | | | | | 1 | | | | | | | | 8 | | | | | | | | | | | | | 9 | | |  |
|  | 214_5 | Do your hands shake? | | | | 0 | | | | | | | | | | | 1 | | | | | | | | 8 | | | | | | | | | | | | | 9 | | |  |
|  | 214_6 | Do you feel nervous, tense or worried? | | | | 0 | | | | | | | | | | | 1 | | | | | | | | 8 | | | | | | | | | | | | | 9 | | |  |
|  | 214_7 | Is your digestion poor? | | | | 0 | | | | | | | | | | | 1 | | | | | | | | 8 | | | | | | | | | | | | | 9 | | |  |
|  | 214_8 | Do you have trouble thinking clearly? | | | | 0 | | | | | | | | | | | 1 | | | | | | | | 8 | | | | | | | | | | | | | 9 | | |  |
|  | 214_9 | Do you feel unhappy? | | | | 0 | | | | | | | | | | | 1 | | | | | | | | 8 | | | | | | | | | | | | | 9 | | |  |
|  | 214_10 | Do you cry more than usual? | | | | 0 | | | | | | | | | | | 1 | | | | | | | | 8 | | | | | | | | | | | | | 9 | | |  |
|  | 214_11 | Do you find it difficult to enjoy your daily activities? | | | | 0 | | | | | | | | | | | 1 | | | | | | | | 8 | | | | | | | | | | | | | 9 | | |  |
|  | 214_12 | Do you find it difficult to make decisions? | | | | 0 | | | | | | | | | | | 1 | | | | | | | | 8 | | | | | | | | | | | | | 9 | | |  |
|  | 214_13 | Is your daily work suffering? | | | | 0 | | | | | | | | | | | 1 | | | | | | | | 8 | | | | | | | | | | | | | 9 | | |  |
|  | 214_14 | Are you unable to play a useful part in life? | | | | 0 | | | | | | | | | | | 1 | | | | | | | | 8 | | | | | | | | | | | | | 9 | | |  |
|  | 214_15 | Have you lost interest in things that you used to enjoy? | | | | 0 | | | | | | | | | | | 1 | | | | | | | | 8 | | | | | | | | | | | | | 9 | | |  |
|  | 214_16 | Do you feel that you are a worthless person? | | | | 0 | | | | | | | | | | | 1 | | | | | | | | 8 | | | | | | | | | | | | | 9 | | |  |
|  | 214_17 | Has the thought of ending your life been on your mind? | | | | | 0 | | | | | | | | | 1 | | | | | | | | 8 | | | | | | | | | | | | | 9 | | | |  |
|  | 214_18 | Do you feel tired all the time? | | | | 0 | | | | | | | | | | | 1 | | | | | | | | 8 | | | | | | | | | | | | | 9 | | |  |
|  | 214_19 | Do you have uncomfortable feelings in your stomach? | | | | 0 | | | | | | | | | | | 1 | | | | | | | | 8 | | | | | | | | | | | | | 9 | | |  |
|  | 214_20 | Are you easily tired? | | | | 0 | | | | | | | | | | | 1 | | | | | | | | 8 | | | | | | | | | | | | | 9 | | |  |
| 215 | Besides the problems that we have already talked about, have you had any of the following symptoms (complaints**) in the past 4 weeks**? If yes, how often? | | | | | | | | | | | | | | | | | | | | | | | | | | | | | | | | | | | | | | | |  |
|  |  | | NEVER | | ONCE OR TWICE PER MONTH | | | | | | | FEW TIMES WEEKLY | | | | | | | | DAILY | | | | | | | | | | | | REFUSED/ NO ANSWER | | | | | | | | |  |
|  | 215_1 | More frequent urination? | 0 | | 1 | | | | | | | 2 | | | | | | | | 3 | | | | | | | | | | | | 9 | | | | | | | | |  |
|  | 215_2 | Thirst? | 0 | | 1 | | | | | | | 2 | | | | | | | | 3 | | | | | | | | | | | | 9 | | | | | | | | |  |
|  | 215_3 | Problems with seeing (visual disturbances) | 0 | | 1 | | | | | | | 2 | | | | | | | | 3 | | | | | | | | | | | | 9 | | | | | | | | |  |
|  | 215_4 | Chest pain? | 0 | | 1 | | | | | | | 2 | | | | | | | | 3 | | | | | | | | | | | | 9 | | | | | | | | |  |
|  | 215_5 | Cough? | 0 | | 1 | | | | | | | 2 | | | | | | | | 3 | | | | | | | | | | | | 9 | | | | | | | | |  |
|  | 215_6 | Dizziness or difficulties in walking straight? | 0 | | 1 | | | | | | | 2 | | | | | | | | 3 | | | | | | | | | | | | 9 | | | | | | | | |  |
|  | 215_7 | Swelling of feet? | 0 | | 1 | | | | | | | 2 | | | | | | | | 3 | | | | | | | | | | | | 9 | | | | | | | | |  |
|  | 215_8 | Numbness of feet? | 0 | | 1 | | | | | | | 2 | | | | | | | | 3 | | | | | | | | | | | | 9 | | | | | | | | |  |
|  | 215_9 | Ulcer on feet? | 0 | | 1 | | | | | | | 2 | | | | | | | | 3 | | | | | | | | | | | | 9 | | | | | | | | |  |
|  | 215_10 | Fever? | 0 | | 1 | | | | | | | 2 | | | | | | | | 3 | | | | | | | | | | | | 9 | | | | | | | | |  |
|  | 215_11 | Excessive sweating | 0 | | 1 | | | | | | | 2 | | | | | | | | 3 | | | | | | | | | | | | 9 | | | | | | | | |  |
|  | 215_12 | Yeast including candida problem and genital fungi infections | 0 | | 1 | | | | | | | 2 | | | | | | | | 3 | | | | | | | | | | | | 9 | | | | | | | | |  |
|  | 215_13 | Unconsciousness | 0 | | 1 | | | | | | | 2 | | | | | | | | 3 | | | | | | | | | | | | 9 | | | | | | | | |  |
|  | 215_14 | Rapid heart rate | 0 | | 1 | | | | | | | 2 | | | | | | | | 3 | | | | | | | | | | | | 9 | | | | | | | | |  |
| 216 | **FOR INTERVIEWER:** MARK ‘YES’ FROM QUESTION 213 WITH X AND ONLY READ UP RELEVANT SYMPTOMS  **If yes** to any of the symptoms above, what did you do to handle the troubles?  Please indicate which symptom and where did you seek help: | | | | | | | | | | | | | | | | | | | | | | | | | | | | | | | | | | | | | | | |  |
|  |  | | | | | SELF-CARE | | | | | ASKED FAMILY/RELATIVES | | | | | | | ASKED NEIGHBORS/  FRIENDS | | | | | BOUGHT MEDICINE | | | | | WENT TO VHW | | | | | WENT TO CHS | | | | | | | WENT TO DISTRIC HOSPITAL |  |
|  | 216a_1 | More frequent urination? | | | | 1 | | | | | 2 | | | | | | | 3 | | | | | 4 | | | | | 5 | | | | | 6 | | | | | | | 7 |  |
|  | 216a_2 | Thirst? | | | | 1 | | | | | 2 | | | | | | | 3 | | | | | 4 | | | | | 5 | | | | | 6 | | | | | | | 7 |  |
|  | 216a_3 | Problems with seeing (visual disturbances) | | | | 1 | | | | | 2 | | | | | | | 3 | | | | | 4 | | | | | 5 | | | | | 6 | | | | | | | 7 |  |
|  | 216a_4 | Chest pain? | | | | 1 | | | | | 2 | | | | | | | 3 | | | | | 4 | | | | | 5 | | | | | 6 | | | | | | | 7 |  |
|  | 216a_5 | Cough? | | | | 1 | | | | | 2 | | | | | | | 3 | | | | | 4 | | | | | 5 | | | | | 6 | | | | | | | 7 |  |
|  | 216a_6 | Dizziness or difficulties in walking straight? | | | | 1 | | | | | 2 | | | | | | | 3 | | | | | 4 | | | | | 5 | | | | | 6 | | | | | | | 7 |  |
|  | 214a_7 | Swelling of feet? | | | | 1 | | | | | 2 | | | | | | | 3 | | | | | 4 | | | | | 5 | | | | | 6 | | | | | | | 7 |  |
|  | 216a_8 | Numbness of feet? | | | | 1 | | | | | 2 | | | | | | | 3 | | | | | 4 | | | | | 5 | | | | | 6 | | | | | | | 7 |  |
|  | 216a_9 | Ulcer on feet? | | | | 1 | | | | | 2 | | | | | | | 3 | | | | | 4 | | | | | 5 | | | | | 6 | | | | | | | 7 |  |
|  | 216a_10 | Fever? | | | | 1 | | | | | 2 | | | | | | | 3 | | | | | 4 | | | | | 5 | | | | | 6 | | | | | | | 7 |  |
|  | 216a_11 | Excessive sweating | | | | 1 | | | | | 2 | | | | | | | 3 | | | | | 4 | | | | | 5 | | | | | 6 | | | | | | | 7 |  |
|  | 216a_12 | Yeast including candida problems? | | | | 1 | | | | | 2 | | | | | | | 3 | | | | | 4 | | | | | 5 | | | | | 6 | | | | | | | 7 |  |
|  | 216a_13 | Unconsciousness | | | | 1 | | | | | 2 | | | | | | | 3 | | | | | 4 | | | | | 5 | | | | | 6 | | | | | | | 7 |  |
|  | 216a_14 | Rapid heart rate | | | | 1 | | | | | 2 | | | | | | | 3 | | | | | 4 | | | | | 5 | | | | | 6 | | | | | | | 7 |  |
| 217 | Has a health care provider informed you on symptoms of **LOW** blood sugar? | | \| NO \| YES \| TO SOME EXTENT \| DON’T KNOW \| REFUSED/ NO ANSWER \| \| --- \| --- \| --- \| --- \| --- \| \| 0 \| 1 \| 2 \| 8 \| 9 \| | | | | | | | | | | | | | | | | | | | | | | | | | | | | | | | | | | | | | |  |
| 217a | Can you feel if your blood sugar is **LOW**? | | \| NO \| YES \| REFUSED/O ANSWER \| \| --- \| --- \| --- \| \| 0 \| 1 \| 9 \| | | | | | | | | | | | | | | | | | | | | | | | | | | | | | | | | | | | | | | If NO  ⇒  216 |
| 217b | If yes, In your perception, how many times **in the last four weeks** have you had a **LOW** **blood sugar (glucose) reaction** with symptoms such as sweating, confusion, headache, fatigue, dizziness, easily angry. | | \| NO \| 0 \| \| --- \| --- \| \| 1-3 TIMES \| 1 \| \| 4 TIMES OR MORE \| 2 \| \| DON’T REMEMBER \| 8 \| \| REFUSED/ NO ANSWER \| 9 \| | | | | | | | | | | | | | | | | | | | | | | | | | | | | | | | | | | | | | |  |
| 218 | Has a healthcare provider informed you on symptoms of **HIGH** blood sugar? | | \| NO \| YES \| TO SOME EXTENT \| REFUSED/ NO ANSWER \| \| --- \| --- \| --- \| --- \| \| 0 \| 1 \| 2 \| 9 \| | | | | | | | | | | | | | | | | | | | | | | | | | | | | | | | | | | | | | |  |
| 218a | Can you feel if your blood sugar is **HIGH**? | | \| NO \| YES \| REFUSED/ NO ANSWER \| \| --- \| --- \| --- \| \| 0 \| 1 \| 9 \| | | | | | | | | | | | | | | | | | | | | | | | | | | | | | | | | | | | | | | If NO  ⇒  217 |
| 218b | If yes, In your perception, how many times **in the last four weeks** have you had a **HIGH blood sugar (glucose) reaction** with symptoms such as increased thirst, frequent peeing, headache, fatigue. | | \| NO \| 0 \| \| --- \| --- \| \| 1-3 TIMES \| 1 \| \| 4 TIMES OR MORE \| 2 \| \| DON’T REMEMBER \| 8 \| \| REFUSED/ NO ANSWER \| 9 \| | | | | | | | | | | | | | | | | | | | | | | | | | | | | | | | | | | | | | |  |
| 219 | I now have some questions about your feelings and experiences with living with diabetes. You can answer on a scale from 1 to 5 where 1 is not a problem and 5 is a serious problem  When answering the questions, please think about how your life has been during **the past four weeks:** | |  | | | | | | | | | | | | | | | | | | | | | | | | | | | | | | | | | | | | | |  |
|  |  | | NOT  A PROBLEM | | | | | MINOR PROBLEM | | | | | | | MODERATE PROBLEM | | | | | | | SOMEWHAT SERIOUS PROBLEM | | | | | | | | SERIOUS PROBLEM | | | | | | | | | DON'T KNOW | |  |
|  |  | | 1 | | | | | 2 | | | | | | | 3 | | | | | | | 4 | | | | | | | | 5 | | | | | | | | | 8 | |  |
|  | 219_1 | Feeling scared when you think about living with diabetes | 1 | | | | | 2 | | | | | | | 3 | | | | | | | 4 | | | | | | | | 6 | | | | | | | | | 8 | |  |
|  | 219_2 | Feeling depressed when you think about living with diabetes | 1 | | | | | 2 | | | | | | | 3 | | | | | | | 4 | | | | | | | | 5 | | | | | | | | | 8 | |  |
|  | 219_3 | Worrying about the future and the possibility of serious compiications | 1 | | | | | 2 | | | | | | | 3 | | | | | | | 4 | | | | | | | | 5 | | | | | | | | | 8 | |  |
|  | 219_4 | Feeling that diabetes is taking up too much of your mental and physical energy every day | 1 | | | | | 2 | | | | | | | 3 | | | | | | | 4 | | | | | | | | 5 | | | | | | | | | 8 | |  |
|  | 219_5 | Coping with complications | 1 | | | | | 2 | | | | | | | 3 | | | | | | | 4 | | | | | | | | 5 | | | | | | | | | 8 | |  |
|  | 219_6 | Having diabetes makes my life difficult financially | 1 | | | | | 2 | | | | | | | 3 | | | | | | | 4 | | | | | | | | 5 | | | | | | | | | 8 | |  |
|  | 219_7 | I am worried that my diabetes will cause my family worries | 1 | | | | | 2 | | | | | | | 3 | | | | | | | 4 | | | | | | | | 5 | | | | | | | | | 8 | |  |
|  | 219_8 | I find that my diabetes is a burden to my family | 1 | | | | | 2 | | | | | | | 3 | | | | | | | 4 | | | | | | | | 5 | | | | | | | | | 8 | |  |
| 220 | Do you try no to think about your diabetes | | \| YES, USUALLY \| 1 \| \| --- \| --- \| \| YES, SOMETIMES \| 2 \| \| RARELY \| 3 \| \| NEVER \| 0 \| \| REFUSED/NO ANSWER \| 9 \| | | | | | | | | | | | | | | | | | | | | | | | | | | | | | | | | | | | | | |  |

| **SECTION 3**  **LIFESTYLE** | | | |
| --- | --- | --- | --- |
| I would now like to ask a few questions about your day-to-day life | | |  |
| 300 | Have you ever smoked? | \| NEVER SMOKED \| SMOKED PREVIOUSLY \| PRESENT SMOKER \| \| --- \| --- \| --- \| \| 0 \| 1 \| 2 \| | If 0, move to 302  If 1, move to 300a  If 2, move to 300b |
| 300a | How many years did you smoke previously? | ______________________ (indicate whole years) | Move to 302 |
| 300b | How many years did you smoke? | ______________________ (indicate whole years) |  |
| 301 | During the past month, how often did you smoke?  (Read up options) | \| In average less than 1 cigarette per day \| 1 \| \| --- \| --- \| \| 1-2 cigarrettes per day \| 2 \| \| 3-10 cigarettes per day \| 3 \| \| 11-20 cigarettes per day \| 4 \| \| More than 20 cigarettes per day \| 5 \| \| DON’T REMEMBER \| 8 \| \| REFUSED/NO ANSWER \| 9 \| |  |
| 302 | During the past month, how often did you drink alcohol?  (Read up options) | \| NEVER \| 0 \| \| --- \| --- \| \| RARELY \| 1 \| \| DAILY OR NEARLY EVERY DAY \| 2 \| \| ONCE OR TWICE A WEEK \| 3 \| \| ONE TO THREE TIMES A MONTH \| 4 \| \| DONT REMEMBER \| 8 \| \| REFUSED/NO ANSWER \| 9 \| |  |
| 303 | During the last month, how often did you exercise (high pulse more than 30 minutes per day)? | \| NEVER \| 0 \| \| --- \| --- \| \| RARELY \| 1 \| \| DAILY OR NEARLY EVERY DAY \| 2 \| \| ONCE OR TWICE A WEEK \| 3 \| \| ONE TO THREE TIMES A MONTH \| 4 \| \| REFUSED/NO ANSWER \| 5 \| |  |

| **SECTION 4**  **“HOMEWORK”** | | | | | | | |
| --- | --- | --- | --- | --- | --- | --- | --- |
| I would now like to ask a few questions about what your health care provider (nurse/doctor) has told you about how to manage your diabetes at home. The questions concern your diet, exercise, medication and blood sugar management. | | | | | | | |
| 401 | Has a health provider given you recommendations regarding what kind of food to eat in your day-to-day life? | | \| NO \| YES \| TO SOME EXTENT \| REFUSED/ NO ANSWER \| \| --- \| --- \| --- \| --- \| \| 0 \| 1 \| 2 \| 9 \| | | | | **IF NO**  ⇒  402 |
| 401a | To which extent is this dietary advice compatible with your daily life? | | \| EASILY \| 1 \| \| --- \| --- \| \| WITH DIFFICULTY \| 2 \| \| SOMETIMES EASILY, SOMETIMES WITH DIFFICULTY \| 3 \| \| REFUSED/ NO ANSWER \| 9 \| | | | |  |
| 401b | In your everyday life, who helps you to follow the nurse/doctor’s recommendations regarding your diet?  (More options allowed) | | \|  \|  \| NO \| YES \| REFUSED \| \| --- \| --- \| --- \| --- \| --- \| \| 401b_1 \| MYSELF \| 0 \| 1 \| 9 \| \| 401b_2 \| HOUSEHOLD MEMBER(S) \| 0 \| 1 \| 9 \| \| 401b_3 \| COMMUNITY MEMBERS \| 0 \| 1 \| 9 \| \| 401b_4 \| HEALTH PROVIDER(S) \| 0 \| 1 \| 9 \| \| 401b_5 \| OTHERS \| 0 \| 1 \| 9 \|   401b_5 If others, specify | | | |  |
| 402 | Has a health provider given you recommendations regarding exercise? | | \| NO \| YES \| TO SOME EXTENT \| REFUSED/ NO ANSWER \| \| --- \| --- \| --- \| --- \| \| 0 \| 1 \| 2 \| 9 \| | | | | **If NO**  ⇒  403 |
| 402a | To which extent is this exercise advice compatible with your daily life? | | \| EASILY \| 1 \| \| --- \| --- \| \| WITH DIFFICULTY \| 2 \| \| SOMETIMES EASILY, SOMETIMES WITH DIFFICULTY \| 3 \| \| REFUSED/ NO ANSWER \| 9 \| | | | |  |
| 402b | In your everyday life, who helps you to follow the nurse/doctor’s recommendations regarding your exercise?  (More options allowed) | | \|  \| \| NO \| YES \| REFUSED \| \| --- \| --- \| --- \| --- \| --- \| \| 402b_1 \| MYSELF \| 0 \| 1 \| 9 \| \| 402b_2 \| HOUSEHOLD MEMBER(S) \| 0 \| 1 \| 9 \| \| 402b_3 \| COMMUNITY MEMBERS \| 0 \| 1 \| 9 \| \| 402b_4 \| HEALTH PROVIDER(S) \| 0 \| 1 \| 9 \| \| 402b_5 \| OTHERS \| 0 \| 1 \| 9 \|   402b_5a: IF OTHERS, SPECIFY:________________________ | | | |  |
| 403 | Has a health provider given you recommendations regarding smoking? | | \| NO \| YES \| TO SOME EXTENT \| REFUSED/ NO ANSWER \| \| --- \| --- \| --- \| --- \| \| 0 \| 1 \| 2 \| 9 \| | | | | **IF NO**  ⇒  404 |
| 403a | To which extent is this advice compatible with your daily life? | | \| I NEVER SMOKED \| 0 \| \| --- \| --- \| \| I STOPPED SMOKING \| 1 \| \| I HAVE DECREASED HOW MUCH I SMOKE \| 2 \| \| I TRIED TO STOP SMOKING, BUT I’M NOW SMOKING AGAIN \| 3 \| \| I DO NOT BELIEVE SMOKING WILL INFLUENCE MY DIABETES \| 4 \| \| DON’T KNOW \| 8 \| \| REFUSED/NO ANSWER \| 9 \| | | | |  |
| 403b | In your everyday life, who helps you to follow the nurse/doctor’s recommendations regarding smoking?  (More options allowed) | | \|  \| \| NO \| YES \| REFUSED \| \| --- \| --- \| --- \| --- \| --- \| \| 403b_1 \| MYSELF \| 0 \| 1 \| 9 \| \| 403b_2 \| HOUSEHOLD MEMBER(S) \| 0 \| 1 \| 9 \| \| 403b_3 \| COMMUNITY MEMBERS \| 0 \| 1 \| 9 \| \| 403b_4 \| HEALTH PROVIDER(S) \| 0 \| 1 \| 9 \| \| 403b_5 \| OTHERS \| 0 \| 1 \| 9 \|   403b_5a: IF OTHERS, SPECIFY:________________________ | | | |  |
| 404 | Has a health provider given you recommendations regarding alcohol? | | \| NO \| YES \| TO SOME EXTENT \| REFUSED/ NO ANSWER \| \| --- \| --- \| --- \| --- \| \| 0 \| 1 \| 2 \| 9 \| | | | | **IF NO**  ⇒  405 |
| 404a | To which extent is this advice compatible with your daily life? | | \| EASILY \| 1 \| \| --- \| --- \| \| WITH DIFFICULTY \| 2 \| \| SOMETIMES EASILY, SOMETIMES WITH DIFFICULTY \| 3 \| \| REFUSED/ NO ANSWER \| 9 \| | | | |  |
| 404b | In your everyday life, who helps you to follow the nurse/doctor’s recommendations regarding alcohol? | | \|  \| \| NO \| YES \| REFUSED \| \| --- \| --- \| --- \| --- \| --- \| \| 404b_1 \| NO ONE \| 0 \| 1 \| 9 \| \| 404b_2 \| HOUSEHOLD MEMBER(S) \| 0 \| 1 \| 9 \| \| 404b_3 \| COMMUNITY MEMBERS \| 0 \| 1 \| 9 \| \| 404b_4 \| HEALTH PROVIDER(S) \| 0 \| 1 \| 9 \| \| 404b_5 \| OTHERS \| 0 \| 1 \| 9 \|   404b_5a: IF OTHERS, SPECIFY:________________________ | | | |  |
| 405 | Has a health provider given you recommendations regarding how to take your medication? | | \| NO \| YES \| TO SOME EXTENT \| REFUSED/ NO ANSWER \| \| --- \| --- \| --- \| --- \| \| 0 \| 1 \| 2 \| 9 \| | | | |  |
| 405a | To which extent is this advice compatible with your daily life? | | \| EASILY \| 1 \| \| --- \| --- \| \| WITH DIFFICULTY \| 2 \| \| SOMETIMES EASILY, SOMETIMES WITH DIFFICULTY \| 3 \| \| REFUSED/ NO ANSWER \| 9 \| | | | |  |
| 405b | In your everyday life, who helps you to follow the nurse/doctor’s recommendations regarding your medication scheme? | | \|  \| \| NO \| YES \| REFUSED \| \| --- \| --- \| --- \| --- \| --- \| \| 405b_1 \| NO ONE \| 0 \| 1 \| 9 \| \| 405b_2 \| HOUSEHOLD MEMBER(S) \| 0 \| 1 \| 9 \| \| 405b_3 \| COMMUNITY MEMBERS \| 0 \| 1 \| 9 \| \| 405b_4 \| HEALTH PROVIDER(S) \| 0 \| 1 \| 9 \| \| 405b_5 \| OTHERS \| 0 \| 1 \| 9 \|   402b_5a: IF OTHERS, SPECIFY:________________________ | | | |  |
| 406 | Has a health care provider informed you on possible negative effects of your medication? | | \| NO \| YES \| TO SOME EXTENT \| REFUSED/ NO ANSWER \| \| --- \| --- \| --- \| --- \| \| 0 \| 1 \| 2 \| 9 \| | | | |  |
| 407 | Has a health care provider informed you on possible complications from diabetes? | | \| NO \| YES \| TO SOME EXTENT \| REFUSED/ NO ANSWER \| \| --- \| --- \| --- \| --- \| \| 0 \| 1 \| 2 \| 9 \| | | | | IF NO  ->408 |
| 407a | What diabetes complications has your health care provider told you about? | |  | | | |  |
|  |  |  | NO | YES |  | REFUSED/NO ANSWER |  |
|  | 407a_1 | HYPERTENSION | 0 | 1 |  | 9 |  |
|  | 407a_2 | SEXUAL PROBLEMS | 0 | 1 |  | 9 |  |
|  | 407a_3 | EYE DAMAGE | 0 | 1 |  | 9 |  |
|  | 407a_4 | FOOT DAMAGE | 0 | 1 |  | 9 |  |
|  | 407a_5 | NERVE DAMAGE | 0 | 1 |  | 9 |  |
|  | 407a_6 | KIDNEY DAMAGE | 0 | 1 |  | 9 |  |
|  | 407a_7 | SKIN CONDITIONS | 0 | 1 |  | 9 |  |
|  | 407a_8 | HEARING PROBLEMS | 0 | 1 |  | 9 |  |
|  | 407a_9 | DEMENTIA | 0 | 1 |  | 9 |  |
|  | 407a_10 | DEPRESSION | 0 | 1 |  | 9 |  |
|  | 407a_11 | CARDIO-VASCULAR DISEASE | 0 | 1 |  | 9 |  |
|  | 407a_12 | OTHER | 0 | 1 |  | 9 |  |
|  |  | 407a_12a: IF OTHERS, SPECIFY:________________________ | | | | |  |
| 408 | What would you like to know more about in relation to your diabetes | | \|  \|  \| NO \| YES \| \| --- \| --- \| --- \| --- \| \| 408_1 \| Medication \| 0 \| 1 \| \| 408_2 \| Diet \| 0 \| 1 \| \| 408_3 \| Exercise \| 0 \| 1 \| \| 408_4 \| Foot care \| 0 \| 1 \| \| 408_5 \| Complications \| 0 \| 1 \| \| 408_6 \| Symptoms of hypoglycemia \| 0 \| 1 \| \| 408_7 \| Symptoms of hyperglycemia \| 0 \| 1 \| | | | |  |

| **SECTION 5**  **INFORMAL SOCIAL SUPPORT** | | | | | | | | | | | |
| --- | --- | --- | --- | --- | --- | --- | --- | --- | --- | --- | --- |
| I will now ask some questions regarding who supports you handling your diabetes. | | | | | | | | | | | |
| 500 | | Do you have other people in your social network who have diabetes? | | \| NO \| YES \| REFUSED/NO ANSWER \| \| --- \| --- \| --- \| \| 0 \| 1 \| 9 \| | | | | | | | **IF NO** ⇒  501 |
| 500a | | Who?  (multiple answering options are allowed) | | \|  \|  \| NO \| YES \| N/A \| \| --- \| --- \| --- \| --- \| --- \| \| 500a_1 \| SPOUSE \| 0 \| 1 \| 8 \| \| 500a_2 \| MOTHER \| 0 \| 1 \| 8 \| \| 500a_3 \| FATHER \| 0 \| 1 \| 8 \| \| 500a_4 \| MOTHER-IN-LAW \| 0 \| 1 \| 8 \| \| 500a_5 \| FATHER-IN-LAW \| 0 \| 1 \| 8 \| \| 500a_6 \| SISTER \| 0 \| 1 \| 8 \| \| 500a_7 \| BROTHER \| 0 \| 1 \| 8 \| \| 500a_8 \| SISTER-IN-LAW \| 0 \| 1 \| 8 \| \| 500a_9 \| BROTHER-IN-LAW \| 0 \| 1 \| 8 \| \| 500a_10 \| DAUGHTER \| 0 \| 1 \| 8 \| \| 500a_11 \| SON \| 0 \| 1 \| 8 \| \| 500a_12 \| DAUGHTER-IN-LAW \| 0 \| 1 \| 8 \| \| 500a_13 \| SON-IN-LAW \| 0 \| 1 \| 8 \| \| 500a_14 \| GRANDCHILD \| 0 \| 1 \| 8 \| \| 500a_15 \| FRIEND \| 0 \| 1 \| 8 \| \| 500a_16 \| NEIGHBOR \| 0 \| 1 \| 8 \| \| 500a_17 \| OTHER \| 0 \| 1 \| 8 \|   501_1a: IF OTHER, SPECIFY: __________________________ | | | | | | |  |
| 501 | Who accompanied you to the health facility when you were diagnosed with diabetes?  (multiple answering options are allowed) | | | \|  \|  \| NO \| YES \| N/A \| \| --- \| --- \| --- \| --- \| --- \| \| 501_1 \| I WAS ALONE \| 0 \| 1 \| 8 \| \| 501_2 \| SPOUSE \| 0 \| 1 \| 8 \| \| 501_3 \| MOTHER \| 0 \| 1 \| 8 \| \| 501_4 \| FATHER \| 0 \| 1 \| 8 \| \| 501_5 \| MOTHER-IN-LAW \| 0 \| 1 \| 8 \| \| 501_6 \| FATHER-IN-LAW \| 0 \| 1 \| 8 \| \| 501_7 \| SISTER \| 0 \| 1 \| 8 \| \| 501_8 \| BROTHER \| 0 \| 1 \| 8 \| \| 501_9 \| SISTER-IN-LAW \| 0 \| 1 \| 8 \| \| 501_10 \| BROTHER-IN-LAW \| 0 \| 1 \| 8 \| \| 501_11 \| DAUGHTER \| 0 \| 1 \| 8 \| \| 501_12 \| SON \| 0 \| 1 \| 8 \| \| 501_13 \| DAUGHTER-IN-LAW \| 0 \| 1 \| 8 \| \| 501_14 \| SON-IN-LAW \| 0 \| 1 \| 8 \| \| 501_15 \| GRANDCHILD \| 0 \| 1 \| 8 \| \| 501_16 \| FRIEND \| 0 \| 1 \| 8 \| \| 501_17 \| NEIGHBOR \| 0 \| 1 \| 8 \| \| 501_18 \| OTHER PERSON WITH DIABETES \| 0 \| 1 \| 8 \| \| 501_19 \| OTHER \| 0 \| 1 \| 8 \|   501_19a: IF OTHER, SPECIFY: _____________________________ | | | | | | |  |
| 502 | Who usually goes to the hospital for the regular treatments with you?  (multiple answering options are allowed) | | | \|  \|  \| NO \| YES \| N/A \| \| --- \| --- \| --- \| --- \| --- \| \| 502_1 \| I GO BY MYSELF \| 0 \| 1 \| 8 \| \| 502_2 \| SPOUSE \| 0 \| 1 \| 8 \| \| 502_3 \| MOTHER \| 0 \| 1 \| 8 \| \| 502_4 \| FATHER \| 0 \| 1 \| 8 \| \| 502_5 \| MOTHER-IN-LAW \| 0 \| 1 \| 8 \| \| 502_6 \| FATHER-IN-LAW \| 0 \| 1 \| 8 \| \| 502_7 \| SISTER \| 0 \| 1 \| 8 \| \| 502_8 \| BROTHER \| 0 \| 1 \| 8 \| \| 502_9 \| SISTER-IN-LAW \| 0 \| 1 \| 8 \| \| 502_10 \| BROTHER-IN-LAW \| 0 \| 1 \| 8 \| \| 502_11 \| DAUGHTER \| 0 \| 1 \| 8 \| \| 502_12 \| SON \| 0 \| 1 \| 8 \| \| 502_13 \| DAUGHTER-IN-LAW \| 0 \| 1 \| 8 \| \| 502_14 \| SON-IN-LAW \| 0 \| 1 \| 8 \| \| 502_15 \| GRANDCHILD \| 0 \| 1 \| 8 \| \| 502_16 \| FRIEND \| 0 \| 1 \| 8 \| \| 502_17 \| NEIGHBOR \| 0 \| 1 \| 8 \| \| 502_18 \| OTHER PERSON WITH DIABETES \| 0 \| 1 \| 8 \| \| 502_19 \| OTHER \| 0 \| 1 \| 8 \|   502_19a: IF OTHER, SPECIFY: __________________________ | | | | | | |  |
| 503 | When you go for your diabetes treatment at the hospital/health centre, how do you **usually** get there? | | | \|  \|  \| NO \| Yes \| REFUSED/NO ANSWER \| \| --- \| --- \| --- \| --- \| --- \| \| 503_1 \| WALK \| 0 \| 1 \| 9 \| \| 503_2 \| BIKE \| 0 \| 1 \| 9 \| \| 503_3 \| MOTOR BIKE \| 0 \| 1 \| 9 \| \| 503_4 \| BUS \| 0 \| 1 \| 9 \| \| 503_5 \| Taxi \| 0 \| 1 \| 9 \| \| 503_6 \| OTHER VEHICLE \| 0 \| 1 \| 9 \|   503_5a: IF OTHER, SPECIFY: _________________________ | | | | | | | IF ONLY WALK  ⇒  505 |
| 504 | Does the transport require payment? | | | \| NO \| YES \| DONT KNOW \| REFUSED/NO ANSWER \| \| --- \| --- \| --- \| --- \| \| 0 \| 1 \| 8 \| 9 \| | | | | | | | IF NO  ⇒  504b |
| 504a | IF YES, who usually pays? | | | \|  \|  \| NO \| YES \| N/A \| \| --- \| --- \| --- \| --- \| --- \| \| 504a_1 \| I PAY MYSELF \| 0 \| 1 \| 8 \| \| 504a_2 \| SPOUSE \| 0 \| 1 \| 8 \| \| 504a_3 \| MOTHER \| 0 \| 1 \| 8 \| \| 504a_4 \| FATHER \| 0 \| 1 \| 8 \| \| 504a_5 \| MOTHER-IN-LAW \| 0 \| 1 \| 8 \| \| 504a_6 \| FATHER-IN-LAW \| 0 \| 1 \| 8 \| \| 504a_7 \| SISTER \| 0 \| 1 \| 8 \| \| 504a_8 \| BROTHER \| 0 \| 1 \| 8 \| \| 504a_9 \| SISTER-IN-LAW \| 0 \| 1 \| 8 \| \| 504a_10 \| BROTHER-IN-LAW \| 0 \| 1 \| 8 \| \| 504a_11 \| DAUGHTER \| 0 \| 1 \| 8 \| \| 504a_12 \| SON \| 0 \| 1 \| 8 \| \| 504a_13 \| DAUGHTER-IN-LAW \| 0 \| 1 \| 8 \| \| 504a_14 \| SON-IN-LAW \| 0 \| 1 \| 8 \| \| 504a_15 \| GRANDCHILD \| 0 \| 1 \| 8 \| \| 504a_16 \| FRIEND \| 0 \| 1 \| 8 \| \| 504a_17 \| NEIGHBOR \| 0 \| 1 \| 8 \| \| 504a_18 \| OTHER \| 0 \| 1 \| 8 \|   504a_18a: IF OTHER, SPECIFY: ________________________ | | | | | | |  |
| 504b | IF NO:  Who **usually** drives you? | | | \|  \|  \| NO \| YES \| N/A \| \| --- \| --- \| --- \| --- \| --- \| \| 504b_1 \| I GO BY MYSELF \| 0 \| 1 \| 8 \| \| 504b_2 \| SPOUSE \| 0 \| 1 \| 8 \| \| 504b_3 \| MOTHER \| 0 \| 1 \| 8 \| \| 504b_4 \| FATHER \| 0 \| 1 \| 8 \| \| 504b_5 \| MOTHER-IN-LAW \| 0 \| 1 \| 8 \| \| 504b_6 \| FATHER-IN-LAW \| 0 \| 1 \| 8 \| \| 504b_7 \| SISTER \| 0 \| 1 \| 8 \| \| 504b_8 \| BROTHER \| 0 \| 1 \| 8 \| \| 504b_9 \| SISTER-IN-LAW \| 0 \| 1 \| 8 \| \| 504b_10 \| BROTHER-IN-LAW \| 0 \| 1 \| 8 \| \| 504b_11 \| DAUGHTER \| 0 \| 1 \| 8 \| \| 504b_12 \| SON \| 0 \| 1 \| 8 \| \| 504b_13 \| DAUGHTER-IN-LAW \| 0 \| 1 \| 8 \| \| 504b_14 \| SON-IN-LAW \| 0 \| 1 \| 8 \| \| 504b_15 \| GRANDCHILD \| 0 \| 1 \| 8 \| \| 504b_16 \| FRIEND \| 0 \| 1 \| 8 \| \| 504b_17 \| NEIGHBOR \| 0 \| 1 \| 8 \| \| 504b_18 \| OTHER PERSON WITH DIABETES \| 0 \| 1 \| 8 \| \| 504b_19 \| OTHER \| 0 \| 1 \| 8 \|   504b_19a: IF OTHER, SPECIFY: ________________________ | | | | | | |  |
| 505 | Have you ever been admitted to hospital overnight due to your diabetes? | | | \| NO \| YES \| REFUSED/NO ANSWER \| \| --- \| --- \| --- \| \| 0 \| 1 \| 9 \| | | | | | | | IF NO  ⇒  506 |
| 505a | IF YES:  Who accompanied you while you were admitted?  (Multiple answers allowed) | | | \|  \|  \| NO \| YES \| N/A \| \| --- \| --- \| --- \| --- \| --- \| \| 505a_1 \| I GO BY MYSELF \| 0 \| 1 \| 8 \| \| 505a_2 \| SPOUSE \| 0 \| 1 \| 8 \| \| 505a_3 \| MOTHER \| 0 \| 1 \| 8 \| \| 505a_4 \| FATHER \| 0 \| 1 \| 8 \| \| 505a_5 \| MOTHER-IN-LAW \| 0 \| 1 \| 8 \| \| 505a_6 \| FATHER-IN-LAW \| 0 \| 1 \| 8 \| \| 505a_7 \| SISTER \| 0 \| 1 \| 8 \| \| 505a_8 \| BROTHER \| 0 \| 1 \| 8 \| \| 505a_9 \| SISTER-IN-LAW \| 0 \| 1 \| 8 \| \| 505a_10 \| BROTHER-IN-LAW \| 0 \| 1 \| 8 \| \| 505a_11 \| DAUGHTER \| 0 \| 1 \| 8 \| \| 505a_12 \| SON \| 0 \| 1 \| 8 \| \| 505a_13 \| DAUGHTER-IN-LAW \| 0 \| 1 \| 8 \| \| 505a_14 \| SON-IN-LAW \| 0 \| 1 \| 8 \| \| 505a_15 \| GRANDCHILD \| 0 \| 1 \| 8 \| \| 505a_16 \| FRIEND \| 0 \| 1 \| 8 \| \| 505a_17 \| NEIGHBOR \| 0 \| 1 \| 8 \| \| 505a_18 \| OTHER PERSON WITH DIABETES \| 0 \| 1 \| 8 \| \| 505a_19 \| OTHER \| 0 \| 1 \| 8 \|   505a_19a: IF OTHER, SPECIFY: ____________________ | | | | | | |  |
| 506 | Who usually pays for your diabetes medication? | | | \|  \|  \| NO \| YES \| N/A \| \| --- \| --- \| --- \| --- \| --- \| \| 506_0 \| COVERED BY INSURANCE \| 0 \| 1 \| 8 \| \| 506_1 \| I PAY MYSELF \| 0 \| 1 \| 8 \| \| 506_2 \| SPOUSE \| 0 \| 1 \| 8 \| \| 506_3 \| MOTHER \| 0 \| 1 \| 8 \| \| 506_4 \| FATHER \| 0 \| 1 \| 8 \| \| 506_5 \| MOTHER-IN-LAW \| 0 \| 1 \| 8 \| \| 506_6 \| FATHER-IN-LAW \| 0 \| 1 \| 8 \| \| 506_7 \| SISTER \| 0 \| 1 \| 8 \| \| 506_8 \| BROTHER \| 0 \| 1 \| 8 \| \| 506_9 \| SISTER-IN-LAW \| 0 \| 1 \| 8 \| \| 506_10 \| BROTHER-IN-LAW \| 0 \| 1 \| 8 \| \| 506_11 \| DAUGHTER \| 0 \| 1 \| 8 \| \| 506_12 \| SON \| 0 \| 1 \| 8 \| \| 506_13 \| DAUGHTER-IN-LAW \| 0 \| 1 \| 8 \| \| 506_14 \| SON-IN-LAW \| 0 \| 1 \| 8 \| \| 506_15 \| GRANDCHILD \| 0 \| 1 \| 8 \| \| 506_16 \| FRIEND \| 0 \| 1 \| 8 \| \| 506_17 \| OTHER \| 0 \| 1 \| 8 \|   506_17a: IF OTHER, SPECIFY: _________________________ | | | | | | |  |
| I will now ask you some questions related to your diabetes when you are **at home.** | | | | | | | | | | |  |
| 507 | Do you have access to a glucometer outside a health facility? | | | \| NO \| YES \| REFUSED/NO ANSWER \| \| --- \| --- \| --- \| \| 0 \| 1 \| 9 \| | | | | | | | IF NO  ⇒  508 |
| 507a | **IF YES**: where? | | | \|  \|  \| NO \| YES \| \| --- \| --- \| --- \| --- \| \| 507a_1 \| IN MY HOUSE \| 0 \| 1 \| \| 507a_2 \| NEIGHBOR \| 0 \| 1 \| \| 507a_3 \| FRIEND \| 0 \| 1 \| \| 507a_4 \| HOUSE OF RELATIVES \| 0 \| 1 \| \| 507a_5 \| OTHER \| 0 \| 1 \|   507a_5a: IF OTHER, SPECIFY: ________________________ | | | | | | |  |
| 507b | Who **usually** measures your blood sugar? | | | \|  \|  \| NO \| YES \| N/A \| \| --- \| --- \| --- \| --- \| --- \| \| 507b_1 \| I DO IT MYSELF \| 0 \| 1 \| 8 \| \| 507b_2 \| SPOUSE \| 0 \| 1 \| 8 \| \| 507b_3 \| MOTHER \| 0 \| 1 \| 8 \| \| 507b_4 \| FATHER \| 0 \| 1 \| 8 \| \| 507b_5 \| MOTHER-IN-LAW \| 0 \| 1 \| 8 \| \| 507b_6 \| FATHER-IN-LAW \| 0 \| 1 \| 8 \| \| 507b_7 \| SISTER \| 0 \| 1 \| 8 \| \| 507b_8 \| BROTHER \| 0 \| 1 \| 8 \| \| 507b_9 \| SISTER-IN-LAW \| 0 \| 1 \| 8 \| \| 507b_10 \| BROTHER-IN-LAW \| 0 \| 1 \| 8 \| \| 507b_11 \| DAUGHTER \| 0 \| 1 \| 8 \| \| 507b_12 \| SON \| 0 \| 1 \| 8 \| \| 507b_13 \| DAUGHTER-IN-LAW \| 0 \| 1 \| 8 \| \| 507b_14 \| SON-IN-LAW \| 0 \| 1 \| 8 \| \| 507b_15 \| GRANDCHILD \| 0 \| 1 \| 8 \| \| 507b_16 \| FRIEND \| 0 \| 1 \| 8 \| \| 507b_17 \| NEIGHBOR \| 0 \| 1 \| 8 \| \| 507b_18 \| OTHER PERSON WITH DIABETES \| 0 \| 1 \| 8 \| \| 507b_19 \| OTHER \| 0 \| 1 \| 8 \|   507b_19a: IF OTHER, SPECIFY: __________________________ | | | | | | |  |
| 508 | Who reminds you of taking your diabetes medication?  (Multiple answers allowed) | | | \|  \|  \| NO \| YES \| N/A \| \| --- \| --- \| --- \| --- \| --- \| \| 508_1 \| I DO IT MYSELF \| 0 \| 1 \| 8 \| \| 508_2 \| SPOUSE \| 0 \| 1 \| 8 \| \| 508_3 \| MOTHER \| 0 \| 1 \| 8 \| \| 508_4 \| FATHER \| 0 \| 1 \| 8 \| \| 508_5 \| MOTHER-IN-LAW \| 0 \| 1 \| 8 \| \| 508_6 \| FATHER-IN-LAW \| 0 \| 1 \| 8 \| \| 508_7 \| SISTER \| 0 \| 1 \| 8 \| \| 508_8 \| BROTHER \| 0 \| 1 \| 8 \| \| 508_9 \| SISTER-IN-LAW \| 0 \| 1 \| 8 \| \| 508_10 \| BROTHER-IN-LAW \| 0 \| 1 \| 8 \| \| 508_11 \| DAUGHTER \| 0 \| 1 \| 8 \| \| 508_12 \| SON \| 0 \| 1 \| 8 \| \| 508_13 \| DAUGHTER-IN-LAW \| 0 \| 1 \| 8 \| \| 508_14 \| SON-IN-LAW \| 0 \| 1 \| 8 \| \| 508_15 \| GRANDCHILD \| 0 \| 1 \| 8 \| \| 508_16 \| FRIEND \| 0 \| 1 \| 8 \| \| 508b_17 \| OTHER PERSON WITH DIABETES \| 0 \| 1 \| 8 \| \| 508_18 \| OTHER \| 0 \| 1 \| 8 \|   508_18a: IF OTHER, SPECIFY: ___________________________ | | | | | | |  |
| 509 | It’s easy to forget to take one’s medication. Does it ever happen to you? | | | Yes 1 No 0 | | | | | | | .  If no, move to 509c |
| 509a | If YES, when this happens to you, will anyone in your household nag you/criticize you for it? | | | \| YES, OFTEN \| 1 \| \| --- \| --- \| \| SOMETIMES \| 2 \| \| RARELY \| 3 \| \| NEVER \| 0 \| \| REFUSED/NO ANSWER \| 9 \| | | | | | | | IF NEVER  ⇒  509c |
| 509b | **IF YES**, who among the following people will nag you/criticize you?  (Multiple answers allowed) | | | \|  \|  \| NO \| YES \| N/A \| \| --- \| --- \| --- \| --- \| --- \| \| 509b_1 \| I GO BY MYSELF \| 0 \| 1 \| 8 \| \| 509b_2 \| SPOUSE \| 0 \| 1 \| 8 \| \| 509b_3 \| MOTHER \| 0 \| 1 \| 8 \| \| 509b_4 \| FATHER \| 0 \| 1 \| 8 \| \| 509b_5 \| MOTHER-IN-LAW \| 0 \| 1 \| 8 \| \| 509b_6 \| FATHER-IN-LAW \| 0 \| 1 \| 8 \| \| 509b_7 \| SISTER \| 0 \| 1 \| 8 \| \| 509b_8 \| BROTHER \| 0 \| 1 \| 8 \| \| 509b_9 \| SISTER-IN-LAW \| 0 \| 1 \| 8 \| \| 509b_10 \| BROTHER-IN-LAW \| 0 \| 1 \| 8 \| \| 509b_11 \| DAUGHTER \| 0 \| 1 \| 8 \| \| 509b_12 \| SON \| 0 \| 1 \| 8 \| \| 509b_13 \| DAUGHTER-IN-LAW \| 0 \| 1 \| 8 \| \| 509b_14 \| SON-IN-LAW \| 0 \| 1 \| 8 \| \| 509b_15 \| GRANDCHILD \| 0 \| 1 \| 8 \| \| 509b_16 \| FRIEND \| 0 \| 1 \| 8 \| \| 509b_17 \| NEIGHBOR \| 0 \| 1 \| 8 \| \| 509b_18 \| OTHER \| 0 \| 1 \| 8 \|   509b_18a: IF OTHER, SPECIFY: _________________________ | | | | | | |  |
| 509c | Have you been told about special care for your feet? | | | \| NO \| YES \| REFUSED/NO ANSWER \| \| --- \| --- \| --- \| \| 0 \| 1 \| 9 \| | | | | | | |  |
| 509d | Does anyone help you to take care of your feet (cutting nails, remove callus)  (Multiple answers allowed) | | | \|  \|  \| NO \| YES \| N/A \| \| --- \| --- \| --- \| --- \| --- \| \| 510b_1 \| MYSELF \| 0 \| 1 \| 8 \| \| 510b_2 \| SPOUSE \| 0 \| 1 \| 8 \| \| 510b_3 \| MOTHER \| 0 \| 1 \| 8 \| \| 510b_4 \| FATHER \| 0 \| 1 \| 8 \| \| 510b_5 \| MOTHER-IN-LAW \| 0 \| 1 \| 8 \| \| 510b_6 \| FATHER-IN-LAW \| 0 \| 1 \| 8 \| \| 510b_7 \| SISTER \| 0 \| 1 \| 8 \| \| 510b_8 \| BROTHER \| 0 \| 1 \| 8 \| \| 510b_9 \| SISTER-IN-LAW \| 0 \| 1 \| 8 \| \| 510b_10 \| BROTHER-IN-LAW \| 0 \| 1 \| 8 \| \| 510b_11 \| DAUGHTER \| 0 \| 1 \| 8 \| \| 510b_12 \| SON \| 0 \| 1 \| 8 \| \| 510b_13 \| DAUGHTER-IN-LAW \| 0 \| 1 \| 8 \| \| 510b_14 \| SON-IN-LAW \| 0 \| 1 \| 8 \| \| 510b_15 \| GRANDCHILD \| 0 \| 1 \| 8 \| \| 510b_16 \| FRIEND \| 0 \| 1 \| 8 \| \| 510b_17 \| OTHER PERSON WITH DIABETES \| 0 \| 1 \| 8 \| \| 510b_18 \| OTHER \| 0 \| 1 \| 8 \|   510b_18a: IF OTHER, SPECIFY: _____________________ | | | | | | |  |
| 510 | Who knows about what you can/cannot eat?  (Multiple answers allowed) | | | \|  \|  \| NO \| YES \| N/A \| \| --- \| --- \| --- \| --- \| --- \| \| 510_1 \| I KNOW IT MYSELF \| 0 \| 1 \| 8 \| \| 510_2 \| SPOUSE \| 0 \| 1 \| 8 \| \| 510_3 \| MOTHER \| 0 \| 1 \| 8 \| \| 510_4 \| FATHER \| 0 \| 1 \| 8 \| \| 510_5 \| MOTHER-IN-LAW \| 0 \| 1 \| 8 \| \| 510_6 \| FATHER-IN-LAW \| 0 \| 1 \| 8 \| \| 510_7 \| SISTER \| 0 \| 1 \| 8 \| \| 510_8 \| BROTHER \| 0 \| 1 \| 8 \| \| 510_9 \| SISTER-IN-LAW \| 0 \| 1 \| 8 \| \| 510_10 \| BROTHER-IN-LAW \| 0 \| 1 \| 8 \| \| 510_11 \| DAUGHTER \| 0 \| 1 \| 8 \| \| 510_12 \| SON \| 0 \| 1 \| 8 \| \| 510_13 \| DAUGHTER-IN-LAW \| 0 \| 1 \| 8 \| \| 510_14 \| SON-IN-LAW \| 0 \| 1 \| 8 \| \| 510_15 \| GRANDCHILD \| 0 \| 1 \| 8 \| \| 510_16 \| FRIEND \| 0 \| 1 \| 8 \| \| 510_17 \| OTHER PERSON WITH DIABETES \| 0 \| 1 \| 8 \| \| 510_18 \| OTHER \| 0 \| 1 \| 8 \|   510_18a: IF OTHER, SPECIFY: __________________________ | | | | | | |  |
| 511 | As compared to other members of your household, do you eat special foods due to your diabetes? | | | \| NO \| YES \| REFUSED/NO ANSWER \| \| --- \| --- \| --- \| \| 0 \| 1 \| 9 \| | | | | | | | IF NO  ⇒  512 |
| 511a | IF YES, who usually prepares this special food?  (Multiple answers allowed) | | | \|  \|  \| NO \| YES \| N/A \| \| --- \| --- \| --- \| --- \| --- \| \| 511a_1 \| I DO IT MYSELF \| 0 \| 1 \| 8 \| \| 511a_2 \| SPOUSE \| 0 \| 1 \| 8 \| \| 511a_3 \| MOTHER \| 0 \| 1 \| 8 \| \| 511a_4 \| FATHER \| 0 \| 1 \| 8 \| \| 511a_5 \| MOTHER-IN-LAW \| 0 \| 1 \| 8 \| \| 511a_6 \| FATHER-IN-LAW \| 0 \| 1 \| 8 \| \| 511a_7 \| SISTER \| 0 \| 1 \| 8 \| \| 511a_8 \| BROTHER \| 0 \| 1 \| 8 \| \| 511a_9 \| SISTER-IN-LAW \| 0 \| 1 \| 8 \| \| 511a_10 \| BROTHER-IN-LAW \| 0 \| 1 \| 8 \| \| 511a_11 \| DAUGHTER \| 0 \| 1 \| 8 \| \| 511a_12 \| SON \| 0 \| 1 \| 8 \| \| 511a_13 \| DAUGHTER-IN-LAW \| 0 \| 1 \| 8 \| \| 511a_14 \| SON-IN-LAW \| 0 \| 1 \| 8 \| \| 511a_15 \| GRANDCHILD \| 0 \| 1 \| 8 \| \| 511a_16 \| FRIEND \| 0 \| 1 \| 8 \| \| 511a_17 \| OTHER \| 0 \| 1 \| 8 \|   511a_17a: IF OTHER, SPECIFY: __________________________ | | | | | | |  |
| 511b | Who usually goes out to purchase the ingredients for the special food that you need due to your diabetes?  Multiple answers allowed | | | \|  \|  \| NO \| YES \| N/A \| \| --- \| --- \| --- \| --- \| --- \| \| 511b_1 \| I DO IT MYSELF \| 0 \| 1 \| 8 \| \| 511b_2 \| SPOUSE \| 0 \| 1 \| 8 \| \| 511b_3 \| MOTHER \| 0 \| 1 \| 8 \| \| 511b_4 \| FATHER \| 0 \| 1 \| 8 \| \| 511b_5 \| MOTHER-IN-LAW \| 0 \| 1 \| 8 \| \| 511b_6 \| FATHER-IN-LAW \| 0 \| 1 \| 8 \| \| 511b_7 \| SISTER \| 0 \| 1 \| 8 \| \| 511b_8 \| BROTHER \| 0 \| 1 \| 8 \| \| 511b_9 \| SISTER-IN-LAW \| 0 \| 1 \| 8 \| \| 511b_10 \| BROTHER-IN-LAW \| 0 \| 1 \| 8 \| \| 511b_11 \| DAUGHTER \| 0 \| 1 \| 8 \| \| 511b_12 \| SON \| 0 \| 1 \| 8 \| \| 511b_13 \| DAUGHTER-IN-LAW \| 0 \| 1 \| 8 \| \| 511b_14 \| SON-IN-LAW \| 0 \| 1 \| 8 \| \| 511b_15 \| GRANDCHILD \| 0 \| 1 \| 8 \| \| 511b_16 \| FRIEND \| 0 \| 1 \| 8 \| \| 511b_17 \| OTHER \| 0 \| 1 \| 8 \|   511b_17a: IF OTHER, SPECIFY: __________________________ | | | | | | |  |
| 511c | Who usually pays for the ingredients for the special food?  (Multiple answers allowed) | | | \|  \|  \| NO \| YES \| N/A \| \| --- \| --- \| --- \| --- \| --- \| \| 511c_1 \| I PAY MYSELF \| 0 \| 1 \| 8 \| \| 511c_2 \| SPOUSE \| 0 \| 1 \| 8 \| \| 511c_3 \| MOTHER \| 0 \| 1 \| 8 \| \| 511c_4 \| FATHER \| 0 \| 1 \| 8 \| \| 511c_5 \| MOTHER-IN-LAW \| 0 \| 1 \| 8 \| \| 511c_6 \| FATHER-IN-LAW \| 0 \| 1 \| 8 \| \| 511c_7 \| SISTER \| 0 \| 1 \| 8 \| \| 511c_8 \| BROTHER \| 0 \| 1 \| 8 \| \| 511c_9 \| SISTER-IN-LAW \| 0 \| 1 \| 8 \| \| 511c_10 \| BROTHER-IN-LAW \| 0 \| 1 \| 8 \| \| 511c_11 \| DAUGHTER \| 0 \| 1 \| 8 \| \| 511c_12 \| SON \| 0 \| 1 \| 8 \| \| 511c_13 \| DAUGHTER-IN-LAW \| 0 \| 1 \| 8 \| \| 511c_14 \| SON-IN-LAW \| 0 \| 1 \| 8 \| \| 511c_15 \| GRANDCHILD \| 0 \| 1 \| 8 \| \| 511c_16 \| FRIEND \| 0 \| 1 \| 8 \| \| 511c_17 \| OTHER \| 0 \| 1 \| 8 \|   511c_17a: IF OTHER, SPECIFY: __________________________ | | | | | | |  |
| 512 | Sometimes people with diabetes will not eat the right food. Does it happen to you? | | | \| NO \| YES \| REFUSED/NO ANSWER \| \| --- \| --- \| --- \| \| 0 \| 1 \| 9 \| | | | | | | | If no, move  to 513 |
| 512a | When you will not eat the right food, do you experience that others will nag/criticize you? | | | \| YES, OFTEN \| 1 \| \| --- \| --- \| \| SOMETIMES \| 2 \| \| RARELY \| 3 \| \| NEVER \| 0 \| \| REFUSED/NO ANSWER \| 9 \| | | | | | | | IF NEVER  ⇒  513 |
| 512b | IF YES, who of the following people will nag/criticize what you eat?  (Multiple answers allowed) | | | \|  \|  \| NO \| YES \| N/A \| \| --- \| --- \| --- \| --- \| --- \| \| 512b_1 \| I WILL BLAME MYSELF \| 0 \| 1 \| 8 \| \| 512b_2 \| SPOUSE \| 0 \| 1 \| 8 \| \| 512b_3 \| MOTHER \| 0 \| 1 \| 8 \| \| 512b_4 \| FATHER \| 0 \| 1 \| 8 \| \| 512b_5 \| MOTHER-IN-LAW \| 0 \| 1 \| 8 \| \| 512b_6 \| FATHER-IN-LAW \| 0 \| 1 \| 8 \| \| 512b_7 \| SISTER \| 0 \| 1 \| 8 \| \| 512b_8 \| BROTHER \| 0 \| 1 \| 8 \| \| 512b_9 \| SISTER-IN-LAW \| 0 \| 1 \| 8 \| \| 512b_10 \| BROTHER-IN-LAW \| 0 \| 1 \| 8 \| \| 512b_11 \| DAUGHTER \| 0 \| 1 \| 8 \| \| 512b_12 \| SON \| 0 \| 1 \| 8 \| \| 512b_13 \| DAUGHTER-IN-LAW \| 0 \| 1 \| 8 \| \| 512b_14 \| SON-IN-LAW \| 0 \| 1 \| 8 \| \| 512b_15 \| GRANDCHILD \| 0 \| 1 \| 8 \| \| 512b_16 \| FRIEND \| 0 \| 1 \| 8 \| \| 512b_17 \| OTHER PERSON WITH DIABETES \| 0 \| 1 \| 8 \| \| 512b_18 \| OTHER \| 0 \| 1 \| 8 \|   512b_18a: IF OTHER, SPECIFY: _________________________ | | | | | | |  |
| 513 | Earlier, you told me that the doctors have told you to exercise for your diabetes.  Who knows that you should exercise?  (Multiple answers allowed) | | | \|  \|  \| NO \| YES \| N/A \| \| --- \| --- \| --- \| --- \| --- \| \| 513_1 \| MYSELF \| 0 \| 1 \| 8 \| \| 513_2 \| SPOUSE \| 0 \| 1 \| 8 \| \| 513_3 \| MOTHER \| 0 \| 1 \| 8 \| \| 513_4 \| FATHER \| 0 \| 1 \| 8 \| \| 513_5 \| MOTHER-IN-LAW \| 0 \| 1 \| 8 \| \| 513_6 \| FATHER-IN-LAW \| 0 \| 1 \| 8 \| \| 513_7 \| SISTER \| 0 \| 1 \| 8 \| \| 513_8 \| BROTHER \| 0 \| 1 \| 8 \| \| 513_9 \| SISTER-IN-LAW \| 0 \| 1 \| 8 \| \| 513_10 \| BROTHER-IN-LAW \| 0 \| 1 \| 8 \| \| 513_11 \| DAUGHTER \| 0 \| 1 \| 8 \| \| 513_12 \| SON \| 0 \| 1 \| 8 \| \| 513_13 \| DAUGHTER-IN-LAW \| 0 \| 1 \| 8 \| \| 513_14 \| SON-IN-LAW \| 0 \| 1 \| 8 \| \| 513_15 \| GRANDCHILD \| 0 \| 1 \| 8 \| \| 513_16 \| FRIEND \| 0 \| 1 \| 8 \| \| 513_17 \| OTHER PERSON WITH DIABETES \| 0 \| 1 \| 8 \| \| 513_18 \| OTHER \| 0 \| 1 \| 8 \|   513_18a: IF OTHER, SPECIFY: __________________________ | | | | | | |  |
| 514 | Does anyone ever remind you about your exercise? | | | \| NO \| YES \| REFUSED/NO ANSWER \| \| --- \| --- \| --- \| \| 0 \| 1 \| 9 \| | | | | | | | IF NO  ⇒  515 |
| 514a | **IF YES**, who?  (Multiple answers allowed) | | | \|  \|  \| NO \| YES \| N/A \| \| --- \| --- \| --- \| --- \| --- \| \| 514a_1 \| MYSELF \| 0 \| 1 \| 8 \| \| 514a_2 \| SPOUSE \| 0 \| 1 \| 8 \| \| 514a_3 \| MOTHER \| 0 \| 1 \| 8 \| \| 514a_4 \| FATHER \| 0 \| 1 \| 8 \| \| 514a_5 \| MOTHER-IN-LAW \| 0 \| 1 \| 8 \| \| 514a_6 \| FATHER-IN-LAW \| 0 \| 1 \| 8 \| \| 514a_7 \| SISTER \| 0 \| 1 \| 8 \| \| 514a_8 \| BROTHER \| 0 \| 1 \| 8 \| \| 514a_9 \| SISTER-IN-LAW \| 0 \| 1 \| 8 \| \| 514a_10 \| BROTHER-IN-LAW \| 0 \| 1 \| 8 \| \| 514a_11 \| DAUGHTER \| 0 \| 1 \| 8 \| \| 514a_12 \| SON \| 0 \| 1 \| 8 \| \| 514a_13 \| DAUGHTER-IN-LAW \| 0 \| 1 \| 8 \| \| 514a_14 \| SON-IN-LAW \| 0 \| 1 \| 8 \| \| 514a_15 \| GRANDCHILD \| 0 \| 1 \| 8 \| \| 514a_16 \| FRIEND \| 0 \| 1 \| 8 \| \| 514a_17 \| OTHER PERSON WITH DIABETES \| 0 \| 1 \| 8 \| \| 514a_18 \| OTHER \| 0 \| 1 \| 8 \|   514a_18a: IF OTHER, SPECIFY: _________________________ | | | | | | |  |
| 515 | Do you usually exercise together with someone? | | | \| NO \| YES \| REFUSED/NO ANSWER \| \| --- \| --- \| --- \| \| 0 \| 1 \| 9 \| | | | | | | | IF NO  ⇒  516 |
| 515a | **IF YES**, who?  (Multiple answers allowed) | | | \|  \|  \| NO \| YES \| N/A \| \| --- \| --- \| --- \| --- \| --- \| \| 515a_1 \| MYSELF \| 0 \| 1 \| 8 \| \| 515a_2 \| SPOUSE \| 0 \| 1 \| 8 \| \| 515a_3 \| MOTHER \| 0 \| 1 \| 8 \| \| 515a_4 \| FATHER \| 0 \| 1 \| 8 \| \| 515a_5 \| MOTHER-IN-LAW \| 0 \| 1 \| 8 \| \| 515a_6 \| FATHER-IN-LAW \| 0 \| 1 \| 8 \| \| 515a_7 \| SISTER \| 0 \| 1 \| 8 \| \| 515a_8 \| BROTHER \| 0 \| 1 \| 8 \| \| 515a_9 \| SISTER-IN-LAW \| 0 \| 1 \| 8 \| \| 515a_10 \| BROTHER-IN-LAW \| 0 \| 1 \| 8 \| \| 515a_11 \| DAUGHTER \| 0 \| 1 \| 8 \| \| 515a_12 \| SON \| 0 \| 1 \| 8 \| \| 515a_13 \| DAUGHTER-IN-LAW \| 0 \| 1 \| 8 \| \| 515a_14 \| SON-IN-LAW \| 0 \| 1 \| 8 \| \| 515a_15 \| GRANDCHILD \| 0 \| 1 \| 8 \| \| 515a_16 \| FRIEND \| 0 \| 1 \| 8 \| \| 515a_17 \| NEIGHBOR \| 0 \| 1 \| 8 \| \| 515a_18 \| OTHER PERSON WITH DIABETES \| 0 \| 1 \| 8 \| \| 515a_19 \| OTHER \| 0 \| 1 \| 8 \|   515a_19a: IF OTHER, SPECIFY: _________________________ | | | | | | |  |
| 516 | Sometimes people with diabetes do not exercise regularly. Does it happen to you? | | | Yes 1 No 0 | | | | | | | If no, move  to 517 |
| 516a | If YES, when it happens to you, do others nag/criticize you? | | | \| YES, OFTEN \| 1 \| \| --- \| --- \| \| SOMETIMES \| 2 \| \| RARELY \| 3 \| \| NEVER \| 0 \| \| REFUSED/NO ANSWER \| 9 \| | | | | | | | IF NEVER  ⇒  517 |
| 516b | **IF YES**, who of the following people will usually nag/criticize you if you don’t exercise?  (Multiple answers allowed) | | | \|  \|  \| NO \| YES \| N/A \| \| --- \| --- \| --- \| --- \| --- \| \| 516b_1 \| MYSELF \| 0 \| 1 \| 8 \| \| 516b_2 \| SPOUSE \| 0 \| 1 \| 8 \| \| 516b_3 \| MOTHER \| 0 \| 1 \| 8 \| \| 516b_4 \| FATHER \| 0 \| 1 \| 8 \| \| 516b_5 \| MOTHER-IN-LAW \| 0 \| 1 \| 8 \| \| 516b_6 \| FATHER-IN-LAW \| 0 \| 1 \| 8 \| \| 516b_7 \| SISTER \| 0 \| 1 \| 8 \| \| 516b_8 \| BROTHER \| 0 \| 1 \| 8 \| \| 516b_9 \| SISTER-IN-LAW \| 0 \| 1 \| 8 \| \| 516b_10 \| BROTHER-IN-LAW \| 0 \| 1 \| 8 \| \| 516b_11 \| DAUGHTER \| 0 \| 1 \| 8 \| \| 516b_12 \| SON \| 0 \| 1 \| 8 \| \| 516b_13 \| DAUGHTER-IN-LAW \| 0 \| 1 \| 8 \| \| 516b_14 \| SON-IN-LAW \| 0 \| 1 \| 8 \| \| 516b_15 \| GRANDCHILD \| 0 \| 1 \| 8 \| \| 516b_16 \| FRIEND \| 0 \| 1 \| 8 \| \| 516b_17 \| NEIGHBOR \| 0 \| 1 \| 8 \| \| 516b_18 \| OTHER PERSON WITH DIABETES \| 0 \| 1 \| 8 \| \| 516b_19 \| OTHER \| 0 \| 1 \| 8 \|   516b_19a: IF OTHER, SPECIFY: ________________________ | | | | | | |  |
| 517 | Who do you usually ask if you have questions or doubts about how to take care of your diabetes?  (Multiple answers allowed) | | | \|  \|  \| NO \| YES \| N/A \| \| --- \| --- \| --- \| --- \| --- \| \| 517_1 \| I FIND INFOMATION \| 0 \| 1 \| 8 \| \| 517_2 \| SPOUSE \| 0 \| 1 \| 8 \| \| 517_3 \| MOTHER \| 0 \| 1 \| 8 \| \| 517_4 \| FATHER \| 0 \| 1 \| 8 \| \| 517_5 \| MOTHER-IN-LAW \| 0 \| 1 \| 8 \| \| 517_6 \| FATHER-IN-LAW \| 0 \| 1 \| 8 \| \| 517_7 \| SISTER \| 0 \| 1 \| 8 \| \| 517_8 \| BROTHER \| 0 \| 1 \| 8 \| \| 517_9 \| SISTER-IN-LAW \| 0 \| 1 \| 8 \| \| 517_10 \| BROTHER-IN-LAW \| 0 \| 1 \| 8 \| \| 517_11 \| DAUGHTER \| 0 \| 1 \| 8 \| \| 517_12 \| SON \| 0 \| 1 \| 8 \| \| 517_13 \| DAUGHTER-IN-LAW \| 0 \| 1 \| 8 \| \| 517_14 \| SON-IN-LAW \| 0 \| 1 \| 8 \| \| 517_15 \| GRANDCHILD \| 0 \| 1 \| 8 \| \| 517_16 \| FRIEND \| 0 \| 1 \| 8 \| \| 517_17 \| HEALTH WORKER \| 0 \| 1 \| 8 \| \| 517_18 \| OTHER PERSON WITH DIABETES \| 0 \| 1 \| 8 \| \| 517_19 \| OTHER \| 0 \| 1 \| 8 \|   517_19a: IF OTHER, SPECIFY: _________________________ | | | | | | |  |
| 518 | Who do you talk to if you are worried about your diabetes?  (Multiple answers allowed) | | | \|  \|  \| NO \| YES \| N/A \| \| --- \| --- \| --- \| --- \| --- \| \| 518_1 \| I MANAGE IT MYSELF \| 0 \| 1 \| 8 \| \| 518_2 \| SPOUSE \| 0 \| 1 \| 8 \| \| 518_3 \| MOTHER \| 0 \| 1 \| 8 \| \| 518_4 \| FATHER \| 0 \| 1 \| 8 \| \| 518_5 \| MOTHER-IN-LAW \| 0 \| 1 \| 8 \| \| 518_6 \| FATHER-IN-LAW \| 0 \| 1 \| 8 \| \| 518_7 \| SISTER \| 0 \| 1 \| 8 \| \| 518_8 \| BROTHER \| 0 \| 1 \| 8 \| \| 518_9 \| SISTER-IN-LAW \| 0 \| 1 \| 8 \| \| 518_10 \| BROTHER-IN-LAW \| 0 \| 1 \| 8 \| \| 518_11 \| DAUGHTER \| 0 \| 1 \| 8 \| \| 518_12 \| SON \| 0 \| 1 \| 8 \| \| 518_13 \| DAUGHTER-IN-LAW \| 0 \| 1 \| 8 \| \| 518_14 \| SON-IN-LAW \| 0 \| 1 \| 8 \| \| 518_15 \| GRANDCHILD \| 0 \| 1 \| 8 \| \| 518_16 \| FRIEND \| 0 \| 1 \| 8 \| \| 518_17 \| HEALTH WORKER \| 0 \| 1 \| 8 \| \| 518_18 \| OTHER PERSON WITH DIABETES \| 0 \| 1 \| 8 \| \| 518_19 \| OTHER \| 0 \| 1 \| 8 \|   518a_19a: IF OTHER, SPECIFY: _________________________ | | | | | | |  |
| 519 | Does anyone encourage or reassure you about your diabetes? | | | \| YES, OFTEN \| 1 \| \| --- \| --- \| \| SOMETIMES \| 2 \| \| RARELY \| 3 \| \| NEVER \| 0 \| \| REFUSED/NO ANSWER \| 9 \| | | | | | | | IF NEVER  ⇒  520 |
| 519a | **IF YES**, who usually encourages or reassures you about your diabetes?  (Multiple answers allowed) | | | \|  \|  \| NO \| YES \| N/A \| \| --- \| --- \| --- \| --- \| --- \| \| 519a_1 \| MYSELF \| 0 \| 1 \| 8 \| \| 519a_2 \| SPOUSE \| 0 \| 1 \| 8 \| \| 519a_3 \| MOTHER \| 0 \| 1 \| 8 \| \| 519a_4 \| FATHER \| 0 \| 1 \| 8 \| \| 519a_5 \| MOTHER-IN-LAW \| 0 \| 1 \| 8 \| \| 519a_6 \| FATHER-IN-LAW \| 0 \| 1 \| 8 \| \| 519a_7 \| SISTER \| 0 \| 1 \| 8 \| \| 519a_8 \| BROTHER \| 0 \| 1 \| 8 \| \| 519a_9 \| SISTER-IN-LAW \| 0 \| 1 \| 8 \| \| 519a_10 \| BROTHER-IN-LAW \| 0 \| 1 \| 8 \| \| 519a_11 \| DAUGHTER \| 0 \| 1 \| 8 \| \| 519a_12 \| SON \| 0 \| 1 \| 8 \| \| 519a_13 \| DAUGHTER-IN-LAW \| 0 \| 1 \| 8 \| \| 519a_14 \| SON-IN-LAW \| 0 \| 1 \| 8 \| \| 519a_15 \| GRANDCHILD \| 0 \| 1 \| 8 \| \| 519a_16 \| FRIEND \| 0 \| 1 \| 8 \| \| 519a_17 \| HEALTH WORKER \| 0 \| 1 \| 8 \| \| 519a_18 \| OTHER PERSON WITH DIABETES \| 0 \| 1 \| 8 \| \| 519a_19 \| OTHER \| 0 \| 1 \| 8 \|   519a_19a: IF OTHER, SPECIFY: _________________________ | | | | | | |  |
| 520 | In case of an emergency with your diabetes, who do you think you could really count on? (Besides health professionals)  (Multiple answers allowed) | | | \|  \|  \| NO \| YES \| N/A \| \| --- \| --- \| --- \| --- \| --- \| \| 520_1 \| MYSELF \| 0 \| 1 \| 8 \| \| 520_2 \| SPOUSE \| 0 \| 1 \| 8 \| \| 520_3 \| MOTHER \| 0 \| 1 \| 8 \| \| 520_4 \| FATHER \| 0 \| 1 \| 8 \| \| 520_5 \| MOTHER-IN-LAW \| 0 \| 1 \| 8 \| \| 520_6 \| FATHER-IN-LAW \| 0 \| 1 \| 8 \| \| 520_7 \| SISTER \| 0 \| 1 \| 8 \| \| 520_8 \| BROTHER \| 0 \| 1 \| 8 \| \| 520_9 \| SISTER-IN-LAW \| 0 \| 1 \| 8 \| \| 520_10 \| BROTHER-IN-LAW \| 0 \| 1 \| 8 \| \| 520_11 \| DAUGHTER \| 0 \| 1 \| 8 \| \| 520_12 \| SON \| 0 \| 1 \| 8 \| \| 520_13 \| DAUGHTER-IN-LAW \| 0 \| 1 \| 8 \| \| 520_14 \| SON-IN-LAW \| 0 \| 1 \| 8 \| \| 520_15 \| GRANDCHILD \| 0 \| 1 \| 8 \| \| 520_16 \| FRIEND \| 0 \| 1 \| 8 \| \| 520_17 \| OTHER PERSON WITH DIABETES \| 0 \| 1 \| 8 \| \| 520_18 \| OTHER \| 0 \| 1 \| 8 \|   520_18a: IF OTHER, SPECIFY: ___________________________ | | | | | | |  |
| 521 | People with diabetes sometimes feel that others discourage or upset them about their diabetes. Do you ever experience this? | | | \| YES, OFTEN \| 1 \| \| --- \| --- \| \| SOMETIMES \| 2 \| \| RARELY \| 3 \| \| NEVER \| 0 \| \| REFUSED/NO ANSWER \| 9 \| | | | | | | | IF NEVER  ⇒  522 |
| 521a | **IF YES**, who discourages you about your diabetes?  (Multiple answers allowed) | | | \|  \|  \| NO \| YES \| N/A \| \| --- \| --- \| --- \| --- \| --- \| \| 521a_1 \| MYSELF \| 0 \| 1 \| 8 \| \| 521a_2 \| SPOUSE \| 0 \| 1 \| 8 \| \| 521a_3 \| MOTHER \| 0 \| 1 \| 8 \| \| 521a_4 \| FATHER \| 0 \| 1 \| 8 \| \| 521a_5 \| MOTHER-IN-LAW \| 0 \| 1 \| 8 \| \| 521a_6 \| FATHER-IN-LAW \| 0 \| 1 \| 8 \| \| 521a_7 \| SISTER \| 0 \| 1 \| 8 \| \| 521a_8 \| BROTHER \| 0 \| 1 \| 8 \| \| 521a_9 \| SISTER-IN-LAW \| 0 \| 1 \| 8 \| \| 521a_10 \| BROTHER-IN-LAW \| 0 \| 1 \| 8 \| \| 521a_11 \| DAUGHTER \| 0 \| 1 \| 8 \| \| 521a_12 \| SON \| 0 \| 1 \| 8 \| \| 521a_13 \| DAUGHTER-IN-LAW \| 0 \| 1 \| 8 \| \| 521a_14 \| SON-IN-LAW \| 0 \| 1 \| 8 \| \| 521a_15 \| GRANDCHILD \| 0 \| 1 \| 8 \| \| 521a_16 \| FRIEND \| 0 \| 1 \| 8 \| \| 521a_17 \| NEIGHBOUR \| 0 \| 1 \| 8 \| \| 521a_18 \| OTHER PERSON WITH DIABETES \| 0 \| 1 \| 8 \| \| 521a_19 \| OTHER \| 0 \| 1 \| 8 \|   521a_19a: IF OTHER, SPECIFY: _________________________ | | | | | | |  |
| 522 | People with diabetes sometimes feel that their family and friends feel uncomfortable about the disease and the person who has it. Do you ever experience this? | | | \| YES, OFTEN \| 1 \| \| --- \| --- \| \| SOMETIMES \| 2 \| \| RARELY \| 3 \| \| NEVER \| 0 \| \| REFUSED/NO ANSWER \| 9 \| | | | | | | | IF NEVER  ⇒  523 |
| 522a | **IF YES**, who seems to feel uncomfortable about your diabetes?  (Multiple answers allowed) | | | \|  \|  \| NO \| YES \| N/A \| \| --- \| --- \| --- \| --- \| --- \| \| 522a_1 \| MYSELF \| 0 \| 1 \| 8 \| \| 522a_2 \| SPOUSE \| 0 \| 1 \| 8 \| \| 522a_3 \| MOTHER \| 0 \| 1 \| 8 \| \| 522a_4 \| FATHER \| 0 \| 1 \| 8 \| \| 522a_5 \| MOTHER-IN-LAW \| 0 \| 1 \| 8 \| \| 522a_6 \| FATHER-IN-LAW \| 0 \| 1 \| 8 \| \| 522a_7 \| SISTER \| 0 \| 1 \| 8 \| \| 522a_8 \| BROTHER \| 0 \| 1 \| 8 \| \| 522a_9 \| SISTER-IN-LAW \| 0 \| 1 \| 8 \| \| 522a_10 \| BROTHER-IN-LAW \| 0 \| 1 \| 8 \| \| 522a_11 \| DAUGHTER \| 0 \| 1 \| 8 \| \| 522a_12 \| SON \| 0 \| 1 \| 8 \| \| 522a_13 \| DAUGHTER-IN-LAW \| 0 \| 1 \| 8 \| \| 522a_14 \| SON-IN-LAW \| 0 \| 1 \| 8 \| \| 522a_15 \| GRANDCHILD \| 0 \| 1 \| 8 \| \| 522a_16 \| FRIEND \| 0 \| 1 \| 8 \| \| 522a_17 \| OTHER \| 0 \| 1 \| 8 \|   522a_17a: IF OTHER, SPECIFY: __________________________ | | | | | | |  |
| 523 | If you think of all the different kinds of support that we have talked about, how important is each of them for you? | | | | | | | | | |  |
|  |  | | | NOT SO IMPORTANT | IMPORTANT | | VERY IMPORTANT | | REFUSED/ NO ANSWER | |  |
|  | 523_1 | | Health care visits/transportation | 0 | 1 | | 2 | | 9 | |  |
|  | 523_2 | | Remembering medication | 0 | 1 | | 2 | | 9 | |  |
|  | 523_3 | | Purchasing and preparing food | 0 | 1 | | 2 | | 9 | |  |
|  | 523_4 | | Remembering and doing exercise | 0 | 1 | | 2 | | 9 | |  |
|  | 523_5 | | Emotional support | 0 | 1 | | 2 | | 9 | |  |
|  | 523_6 | | Financial support with any of the above | 0 | 1 | | 2 | | 9 | |  |
|  | 523_7 | | Other | 0 | 1 | | 2 | | 9 | |  |
|  |  | | 523a: if other, specify: ……………………………….. | | | | | | | |  |
| 524 | We have now talked about different kinds of support that you receive from people around you, with health care visits, medication, food, exercise, emotional support, and so on. Besides these forms of support, do you receive other kinds of support from family, friends and community members? | | | \| NO \| YES \| REFUSED/NO ANSWER \| \| --- \| --- \| --- \| \| 0 \| 1 \| 9 \| | | | | | | | IF NO  ⇒  526 |
| 524a | IF YES, please specify: | | | ____________________________________________________ | | | | | | |  |
| 525 | Sometimes, people with diabetes feel that they do not receive adequate support from family and friends, or that they receive too much attention from others. In your case, how do you assess the support that you receive from family/friends/community with the following things:  (FOR INTERVIEWER: THESE ARE SENSITIVE QUESTIONS – ASK VERY CAREFULLY) | | |  | | | | | | |  |
|  |  | | | I RECEIVE TOO LITTLE SUPPORT | | I RECEIVE ADEQUATE SUPPORT | | I RECEIVE TOO MUCH SUPPORT | | REFUSED/NO ANSWER |  |
|  | 525_1 | | Health care visits/transportation | 1 | | 2 | | 3 | | 9 |  |
|  | 525_2 | | Remembering medication | 1 | | 2 | | 3 | | 9 |  |
|  | 525_3 | | Purchasing and preparing food | 1 | | 2 | | 3 | | 9 |  |
|  | 525_4 | | Remembering and doing exercise | 1 | | 2 | | 3 | | 9 |  |
|  | 525_5 | | Emotional support | 1 | | 2 | | 3 | | 9 |  |
|  | 525_6 | | Financial support with any of the above | 1 | | 2 | | 3 | | 9 |  |
|  | 525_7 | | Other | 1 | | 2 | | 3 | | 9 |  |
|  |  | | 526a: IF OTHER, PLEASE SPECIFY: ___________________________________________________ | | | | | | | |  |
| 526 | Among the people who are supporting you now that you have diabetes, whom would you define as your **most important** support person?  (Only one anwer allowed)  (If participant is uncertain, probe by reading up options) | | | \|  \|  \| \| --- \| --- \| \| SPOUSE \| 1 \| \| MOTHER \| 2 \| \| FATHER \| 3 \| \| MOTHER-IN-LAW \| 4 \| \| FATHER-IN-LAW \| 5 \| \| SISTER \| 6 \| \| BROTHER \| 7 \| \| SISTER-IN-LAW \| 8 \| \| BROTHER-IN-LAW \| 9 \| \| DAUGHTER \| 10 \| \| SON \| 11 \| \| DAUGHTER-IN-LAW \| 12 \| \| SON-IN-LAW \| 13 \| \| GRANDCHILD \| 14 \| \| FRIEND \| 15 \| \| ANOTHER PERSON WITH DIABETES \| 16 \| \| NOBODY \| 17 \| \| OTHER \| 18 \|   526a: IF OTHER, SPECIFY: ____________________ | | | | | | |  |
| 527 | Among the people who are supporting you, whom would you define as your **second** **most important** support person?  (Only one anwer allowed)  (If participant is uncertain, probe by reading up options) | | | \|  \|  \| \| --- \| --- \| \| MYSELF \| 0 \| \| SPOUSE \| 1 \| \| MOTHER \| 2 \| \| FATHER \| 3 \| \| MOTHER-IN-LAW \| 4 \| \| FATHER-IN-LAW \| 5 \| \| SISTER \| 6 \| \| BROTHER \| 7 \| \| SISTER-IN-LAW \| 8 \| \| BROTHER-IN-LAW \| 9 \| \| DAUGHTER \| 10 \| \| SON \| 11 \| \| DAUGHTER-IN-LAW \| 12 \| \| SON-IN-LAW \| 13 \| \| GRANDCHILD \| 14 \| \| FRIEND \| 15 \| \| ANOTHER PERSON WITH DIABETES \| 16 \| \| NOBODY \| 17 \| \| OTHER \| 18 \|   527a: IF OTHER, SPECIFY: _______________________________ | | | | | | |  |
| 528 | Among the people who are supporting you, whom would you define as your **third** **most important** support person?  (Only one anwer allowed)  (If participant is uncertain, probe by reading up options) | | | \|  \|  \| \| --- \| --- \| \| SPOUSE \| 1 \| \| MOTHER \| 2 \| \| FATHER \| 3 \| \| MOTHER-IN-LAW \| 4 \| \| FATHER-IN-LAW \| 5 \| \| SISTER \| 6 \| \| BROTHER \| 7 \| \| SISTER-IN-LAW \| 8 \| \| BROTHER-IN-LAW \| 9 \| \| DAUGHTER \| 10 \| \| SON \| 11 \| \| DAUGHTER-IN-LAW \| 12 \| \| SON-IN-LAW \| 13 \| \| GRANDCHILD \| 14 \| \| FRIEND \| 15 \| \| ANOTHER PERSON WITH DIABETES \| 16 \| \| NOBODY \| 17 \| \| OTHER \| 18 \|   528a: IF OTHER, SPECIFY: ______________________________ | | | | | | |  |

| **SECTION 6**  **SEXUAL WELL-BEING AND EMOTIONS** | | | |
| --- | --- | --- | --- |
| We have now come to the last part of the interview. I would like to wrap up by asking you a few questions about how diabetes affects your sexual health. More than 50% of people (both women and men) with diabetes experience sexual health problems. Many patients feel that it is difficult to talk to their doctor about this. We would like to ask you a few questions so that we can get more knowledge about diabetes and sexual health. | | | |
| 601 | **During the past four weeks**, how satisfied have you been with your sexual life? | \| NOT AT ALL \| 1 \| \| --- \| --- \| \| MINIMALLY \| 2 \| \| MODERATELY \| 3 \| \| I’M SATISFIED \| 4 \| \| I’M VERY SATISFIED \| 5 \| \| I HAVE NO SEX LIFE \| 6 \| \| REFUSED/NO ANSWER \| 9 \| |  |
| 601a | When was the last time you’ve had sex **(with a partner or alone)?** | \| Less than one week ago \| 1 \| \| --- \| --- \| \| Approximately one month ago \| 2 \| \| Between 1 month and 6 months ago \| 3 \| \| Between 6 months and 1 year ago \| 4 \| \| Between 1 year and 5 years ago \| 5 \| \| More than 5 years ago \| 6 \| \| No answer/refused \| 9 \| |  |
| 602 | If you compare your sexual life now to the sexual life you had before you got diabetes, how would you characterize the difference? | \| NO DIFFERENCE \| 1 \| \| --- \| --- \| \| MORE SATISFYING NOW \| 2 \| \| LESS SATISFYING NOW \| 3 \| \| MUCH LESS SATISFYING NOW \| 4 \| \| I HAVE NO SEX LIFE \| 5 \| \| REFUSED/NO ANSWER \| 9 \| | IF  1, 2  ⇒  605 |
| 603 | If you feel that your sex life is less satisfying now, to which extent is this a problem for you? | \| IT’S A BIG PROBLEM \| 1 \| \| --- \| --- \| \| IT’S A SMALL PROBLEM \| 2 \| \| IT’S NOT A PROBLEM \| 0 \| \| REFUSED/NO ANSWER \| 9 \| |  |
| 604 | Have the sexual problems changed your relationship to your spouse/partner? | \| NO \| YES \| REFUSED/NO ANSWER \| \| --- \| --- \| --- \| \| 0 \| 1 \| 9 \| | If no, move to 605 |
| 604a | IF YES, how has the sexual problems changed your relationship to your spouse/partner? | \| BETTER \| 1 \| \| --- \| --- \| \| WORSE \| 2 \| \| NOT BETTER/WORSE, BUT DIFFERENT \| 3 \| \| REFUSED/NO ANSWER \| 9 \| |  |
| 605 | Do you think your spouse/partner is satisfied with your sexual life? | \| NO \| YES \| NOT REALLY \| REFUSED/  NO ANSWER \| \| --- \| --- \| --- \| --- \| \| 0 \| 1 \| 2 \| 9 \| |  |
| 606 | Have you been getting treatment for this problem? | \| NO \| YES \| REFUSED/NO ANSWER \| \| --- \| --- \| --- \| \| 0 \| 1 \| 9 \| | IF NO  ⇒  607 |
| 606a | IF YES; did the medication work? | \| NO \| YES \| TO SOME EXTENT \| REFUSED/NO ANSWER \| \| --- \| --- \| --- \| --- \| \| 0 \| 1 \| 2 \| 9 \| |  |
| 607 | With whom have you talked about the sexual health problems that you experience?  (Multiple answers allowed) | \|  \|  \| NO \| YES \| N/A \| \| --- \| --- \| --- \| --- \| --- \| \| 607_1 \| SPOUSE \| 0 \| 1 \| 8 \| \| 607_2 \| MOTHER \| 0 \| 1 \| 8 \| \| 607_3 \| FATHER \| 0 \| 1 \| 8 \| \| 607_4 \| MOTHER-IN-LAW \| 0 \| 1 \| 8 \| \| 607_5 \| FATHER-IN-LAW \| 0 \| 1 \| 8 \| \| 607_6 \| SISTER \| 0 \| 1 \| 8 \| \| 607_7 \| BROTHER \| 0 \| 1 \| 8 \| \| 607_8 \| SISTER-IN-LAW \| 0 \| 1 \| 8 \| \| 607_9 \| BROTHER-IN-LAW \| 0 \| 1 \| 8 \| \| 607_10 \| DAUGHTER \| 0 \| 1 \| 8 \| \| 607_11 \| SON \| 0 \| 1 \| 8 \| \| 607_12 \| DAUGHTER-IN-LAW \| 0 \| 1 \| 8 \| \| 607_13 \| SON-IN-LAW \| 0 \| 1 \| 8 \| \| 607_14 \| GRANDCHILD \| 0 \| 1 \| 8 \| \| 607_15 \| HEALTH WORKER \| 0 \| 1 \| 8 \| \| 607_16 \| FRIEND \| 0 \| 1 \| 8 \| \| 607_17 \| NEIGHBOR \| 0 \| 1 \| 8 \| \| 607_18 \| PERSON WITH DIABETES \| 0 \| 1 \| 8 \| \| 607_19 \| NOBODY \| 0 \| 1 \| 8 \| \| 607_20 \| OTHER \| 0 \| 1 \| 8 \|   607_20a: IF OTHER, SPECIFY: _________________________ |  |

| **SECTION 7**  **Use of smart phones, internet and other types of media** | | | |
| --- | --- | --- | --- |
| 700 | What source do you use to obtain information about diabetes?  (multiple answering options are allowed)  (alone or with assistance from others) | \|  \|  \| NO \| YES \| REFUSED/NO ANSWER \| \| --- \| --- \| --- \| --- \| --- \| \| 700_1 \| TELEVISION \| 0 \| 1 \| 9 \| \| 700_2 \| RADIO \| 0 \| 1 \| 9 \| \| 700_3 \| VILLAGE SPEAKER SYSTEM \| 0 \| 1 \| 9 \| \| 700_4 \| NEWSPAPER \| 0 \| 1 \| 9 \| \| 700_5 \| OTHER \| 0 \| 1 \| 9 \|   700a: IF OTHER, SPECIFY:_______________________ |  |
| 701 | Do you have a mobile phone? | \| NO \| YES \| REFUSED/NO ANSWER \| \| --- \| --- \| --- \| \| 0 \| 1 \| 9 \| | If NO  ⇒  702 |
| 701a | If yes, what kind of phone? | \|  \|  \| NO \| YES \| REFUSED/ NO ANSWER \| \| --- \| --- \| --- \| --- \| --- \| \| 701a_1 \| SMARTPHONE \| 0 \| 1 \| 9 \| \| 701a_2 \| MOBILE PHONE WITH PUSH BUTTONS \| 0 \| 1 \| 9 \| |  |
| 702 | Do you access social media (e.g. Facebook, ZALO, LINE) or internet? (alone or with assistance from others) | \| NO \| YES \| REFUSED/NO ANSWER \| \| --- \| --- \| --- \| \| 0 \| 1 \| 9 \| | If NO  ⇒  SECTION 8 |
| 703 | Do you use a smartphone to read or write about diabetes? (alone or with assistance from others) | \| NO \| YES \| REFUSED/NO ANSWER \| \| --- \| --- \| --- \| \| 0 \| 1 \| 9 \| | If NO  ⇒  SECTION 8 |
| 704 | What type (of electronic platform) do you access?  (multiple answering options are allowed)  (alone or with assistance from others) | \|  \|  \| NO \| YES \| REFUSED/NO ANSWER \| \| --- \| --- \| --- \| --- \| --- \| \| 704_1 \| SOCIAL MEDIA \| 0 \| 1 \| 9 \| \| 704_2 \| INTERNET SITES \| 0 \| 1 \| 9 \| \| 704_3 \| OTHER \| 0 \| 1 \| 9 \|   704a: IF OTHER, SPECIFY:_______________________ |  |
| 705 | Do you trust the information about diabetes that you receive through the Internet or social media? | \| NO \| YES \| PARTLY \| REFUSED/NO ANSWER \| \| --- \| --- \| --- \| --- \| \| 0 \| 1 \| 2 \|  \| |  |

| **SECTION 8**  **COMPLETION OF INTERVIEW** | | |
| --- | --- | --- |
|  | We have now finished the interview. I would like to thank you very much for helping us. I appreciate the time that you have taken.  Do you have any comments, or is there anything else you would like to add?  As part of this porject, we will conduct an intervention in two communes to improve the lives for people with diabetes in thai binh province. What do you think would be the most important acivities? |  |
|  |  |  |
|  | In order to learn more about how people living with diabetes can best be supported, we would like to talk to three people that you identified as your most important support persons.  Is it okay with you if we contact them and ask if we can conduct an interview with them?  YES………  NO……….  **IF** **YES**, kindly explain how we can best contact them:  SUPPORT PERSON NO 1: …………  SUPPORT PERSON NO 2:………….  SUPPORT PERSON NO 3:………….. |  |

| INTERVIEWER COMMENTS TO BE COMPLETED AFTER INTERVIEW | | |
| --- | --- | --- |
|  | __________________________________________________________________________________________________________________________________________________________________________________________________________________________________________________________________________________________________________________________________________________________________________________________________________________________________________________________________________________________________________________________________________________________________________________________________________________________________________________________________________________________________________________________________________________________________________________________________________________________________________________________________________________________________________________________________________________________________________________________________________________________________________________________________________________________________________________________________________________________________________________________________________________________________________________________________________________________________________________________________________________________________________________________________________________________________________________________________________________________________________________________________________________________________________________________________________________________________________________________________________________________________________________________________________________________________________________________________________________ |  |
